# The multidisciplinary approach to eosinophilia

**DOI:** 10.3389/fonc.2023.1193730

**Published:** 2023-05-18

**Authors:** Gunhild Nynke Thomsen, Mette Niemann Christoffersen, Hanne Merete Lindegaard, Jesper Rømhild Davidsen, Gitte Nyvang Hartmeyer, Kristian Assing, Charlotte G. Mortz, Raquel Martin-Iguacel, Michael Boe Møller, Anette Drøhse Kjeldsen, Troels Havelund, Daniel El Fassi, Sigurd Broesby-Olsen, Michael Maiborg, Sofie Lock Johansson, Christen Lykkegaard Andersen, Hanne Vestergaard, Ole Weis Bjerrum

**Affiliations:** ^1^ Department of Hematology, Odense University Hospital, Odense, Denmark; ^2^ Department of Rheumatology, Odense University Hospital, Denmark; Research Unit for Rheumatology, Odense University Hospital, Odense, Denmark; University of Southern Denmark, Odense, Denmark; ^3^ Department of Respiratory Medicine, Odense University Hospital, Denmark; Odense Respiratory Research Unit (ODIN), Department of Clinical Research, University of Southern Denmark, Odense, Denmark; ^4^ Department of Clinical Microbiology, Odense University Hospital, Odense, Denmark; ^5^ Department of Clinical Immunology, Odense University Hospital, Odense, Denmark; ^6^ Department of Dermatology and Allergy Centre, Odense Research Centre for Anaphylaxis (ORCA), Odense University Hospital, Denmark; University of Southern Denmark, Odense, Denmark; ^7^ Department of Infectious Diseases , Odense University Hospital, Odense, Denmark; ^8^ Department of Pathology, Odense University Hospital, Odense, Denmark; ^9^ Department of ORL- Head and Neck Surgery and Audiology, Odense University Hospital, Odense, Denmark; University of Southern Denmark, Odense, Denmark; ^10^ Department of Gastroenterology and Hepatology, Odense University Hospital, Odense, Denmark; ^11^ Department of Hematology, Copenhagen University Hospital, Rigshospitalet, Copenhagen, Denmark; ^12^ Department of Clinical Medicine, University of Copenhagen, Copenhagen, Denmark; ^13^ Department of Cardiology, Odense University Hospital, Odense, Denmark; ^14^ Department of Respiratory Medicine, Odense University Hospital, Odense, Denmark; ^15^ Centre for General Practice, Department of Public Health, University of Copenhagen, Copenhagen, Denmark

**Keywords:** clinical, diagnosis, eosinophilia, multidisciplinary, review, trial, treatment

## Abstract

Eosinophilic granulocytes are normally present in low numbers in the bloodstream. Patients with an increased number of eosinophilic granulocytes in the differential count (eosinophilia) are common and can pose a clinical challenge because conditions with eosinophilia occur in all medical specialties. The diagnostic approach must be guided by a thorough medical history, supported by specific tests to guide individualized treatment. Neoplastic (primary) eosinophilia is identified by one of several unique acquired genetic causes. In contrast, reactive (secondary) eosinophilia is associated with a cytokine stimulus in a specific disease, while idiopathic eosinophilia is a diagnosis by exclusion. Rational treatment is disease-directed in secondary cases and has paved the way for targeted treatment against the driver in primary eosinophilia, whereas idiopathic cases are treated as needed by principles in eosinophilia originating from clonal drivers. The vast majority of patients are diagnosed with secondary eosinophilia and are managed by the relevant specialty—e.g., rheumatology, allergy, dermatology, gastroenterology, pulmonary medicine, hematology, or infectious disease. The overlap in symptoms and the risk of irreversible organ involvement in eosinophilia, irrespective of the cause, warrants that patients without a diagnostic clarification or who do not respond to adequate treatment should be referred to a multidisciplinary function anchored in a hematology department for evaluation. This review presents the pathophysiology, manifestations, differential diagnosis, diagnostic workup, and management of (adult) patients with eosinophilia. The purpose is to place eosinophilia in a clinical context, and therefore justify and inspire the establishment of a multidisciplinary team of experts from diagnostic and clinical specialties at the regional level to support the second opinion. The target patient population requires highly specialized laboratory analysis and therapy and occasionally has severe eosinophil-induced organ dysfunction. An added value of a centralized, clinical function is to serve as a platform for education and research to further improve the management of patients with eosinophilia. Primary and idiopathic eosinophilia are key topics in the review, which also address current research and discusses outstanding issues in the field.

## 1 Introduction

The differential count of white blood cells is a simple analysis to obtain diagnostic information, and deviations in the number of leukocytes reflect perturbed homeostasis. Leukocytosis and leukopenia, associated with neutrophilic granulocytes or lymphocytes, are important clues when evaluating a patient, indicating, for example, a feedback control request for immunocompetent cells to fight infections or a derailed leukopoiesis with or without maturation (1, 2). Eosinophilic granulocytes (eosinophils) are normally among the least abundant circulating white blood cells (<0.5 × 10^9^/L). Unlike common leukocytes, a reduced number of eosinophils is not captured by the differential count and is normally not clinically paid attention to. In contrast to this, the observation of an increase in eosinophils in a differential count of blood or other samples can be a key piece of information that should be contextualized in advance in the individual (adult) patient. However, a structured approach is required to guide diagnostic and therapeutic decisions clinically, and the task is to isolate the impact of an increased eosinophil count (eosinophilia) from all other etiologic factors in the overall assessment (3–8).

Chronic myeloproliferative neoplasms (MPNs) include several distinct disorders, representing an autonomous turnover of one or more of the cells, circulating in the blood. MPNs include, according to the current WHO classification, breakpoint cluster region—Abelson1 (*BCR-ABL1*)-positive chronic myeloid leukemia (CML) and the *BCR-ABL1*-negative neoplasms, many of which are considered to be inflammatory conditions driving a clonal evolution in a biological continuum involving variable mutations and genetic structural aberrations (9–14). A separate category in the WHO classification is the myeloid/lymphoid neoplasms with eosinophilia and tyrosine kinase fusion genes (MLN-TK), which are usually prominent features at diagnosis (9).

Primary eosinophilia is rare and reflects clonal hematopoiesis in which the production of eosinophils is driven by a genetic or intrinsic cause. Causes of secondary eosinophilia are common and very different in nature and are characterized as being reactive to factors with an extrinsic impact on the eosinopoiesis. Secondary or reactive eosinophilia is driven, in particular, by the cytokine interleukin (IL)-5, produced by activated T lymphocytes (15, 16). This scenario of immunological crosstalk is associated with autoimmune, infectious, and inflammatory diseases; malignancy; and allergy, including iatrogenic, drug-induced adverse reactions (3–8). When no congenital, clonal, or reactive cause can be demonstrated, patients with persistent eosinophil counts of at least 1.5 × 10^9^/L are categorized as idiopathic hypereosinophilic. This group can be subdivided into patients with no manifestations of eosinophilia [iHE, or hypereosinophilia of undetermined significance (iHE_US_)] or idiopathic hypereosinophilic syndrome (iHES), when organ involvement due to eosinophils is present (3–8, 17–20). However, the overlap of symptoms, regardless of whether the cause of the disease is primary, secondary, or idiopathic eosinophilia—or a combination of them—is considerable, and characterizes the patient with an increased eosinophil count in blood as a clinical challenge.

Notwithstanding the cause, the presence of an increased number of circulating eosinophils in the blood may be associated with inappropriate organ involvement. In most cases, patients with secondary eosinophilia are treated successfully by the general practitioner (GP) or at departments specialized in the management of individual manifestations. However, it can be difficult to prove whether the patient has primary eosinophilia, secondary eosinophilia, or iHES—or whether the symptoms and cause of the eosinophilia can be attributed to an atypical presentation or represent more than one etiology. An insufficient response to symptoms, and unexplained persistent or recurrent eosinophilia despite adequate treatment may be a reason for a thorough reassessment. This review describes eosinophilia in a clinical context, particularly how a dedicated function with a multidisciplinary team is one way to provide a rational approach due to the complexity of demonstrating differential diagnosis and options for targeted treatment. Current unresolved issues in the management of eosinophilia are discussed.

## 2 Review of the eosinophil granulocyte in health and disease

### 2.1 The eosinophil granulocyte and pathophysiology

Eosinophils originate from a myeloid cluster of differentiation (CD) CD34+ precursors in the bone marrow and are part of the innate immune system (16, 21). Maturation takes a week on average and is influenced by a granulocyte-macrophage colony-stimulating factor (GM-CSF), IL3, and IL-5 and is driven by activation of transcription factor networks including PU.1, CCAAT/enhancer-binding protein (C/EBP), and GATA-binding protein 1 (GATA-1). All of the above are involved, but IL-5 and GATA-1 have key roles in eosinopoiesis and turnover; IL-5 is involved in egress from the bone marrow microenvironment into the bloodstream, promoting activation and survival and preventing apoptosis; and GATA-1 is a vital regulator of cell maturation (22–24).

The course of eosinophils subsequent to bone marrow release starts with circulation in the bloodstream for 8–18 h and terminates in peripheral organs for up to 12–14 days or longer (25, 26). At some point during circulation in the bloodstream, the eosinophil migrates through the lining of blood vessels and enters one of the numerous organs, possibly attracted by chemokines (27). Being mobile cells, they are distributed to the liver, lungs, skin, heart, reticuloendothelial system, glands, and digestive tract, but not to the esophagus, which is normally devoid of eosinophils. The cells remain in the organs as tissue-resident cells under homeostatic conditions. During this time, the cell may proliferate under inflammatory conditions and undergo terminal apoptosis (28, 29).

**Figure 1:**
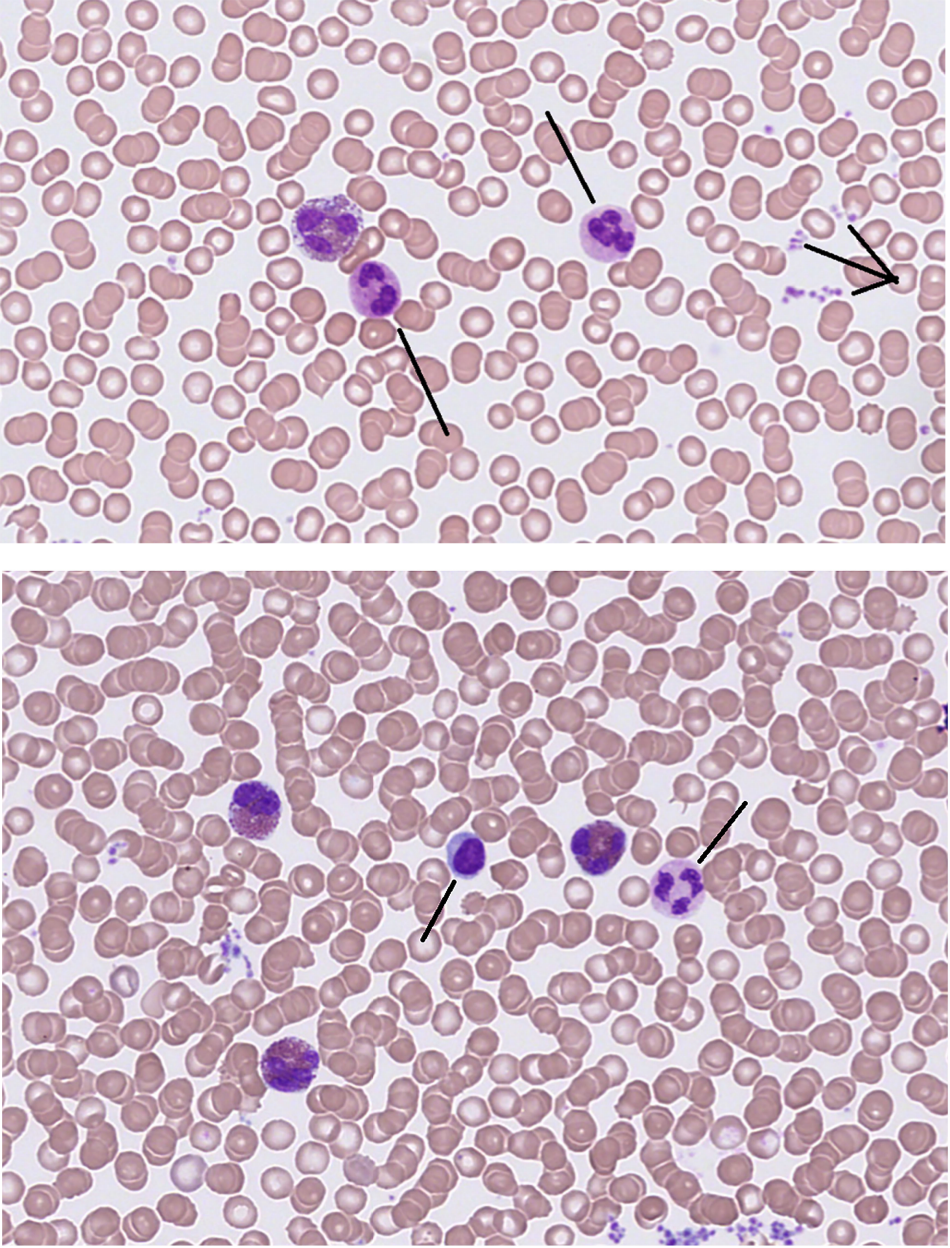
Eosinophil granulocytes in peripheral blood smear. Giemsa stain, 400×. Upper panel illustrates one eosinophil, neutrophil granulocytes (/), and aggregates of platelets (>). Lower panel shows eosinophil granulocytes, a mononuclear cell (/ lymphocyte), a polynuclear granulocyte (/ neutrophil), and thrombocytes (not marked).

Eosinophils in the bloodstream or tissues are large, spherical cells, 12–17 μm in size with a bi-lobed nucleus, without a nucleolus, and exhibiting numerous coarse, rounded, and red-purple granules in the cytoplasm by routine staining (16, 24, 28). **Figure 1** shows peripheral blood smears, illustrating mature eosinophil granulocytes from a patient with mild eosinophilia. Eosinophils can be compared morphologically to neutrophil granulocytes, lymphocytes, and platelets. Typically, less than one eosinophil granulocyte will be recognized in a field using light microscopy. Detection of more than one eosinophil in a 400× light microscopy field examination is an indication of eosinophilia, normally representing 1%–4% of all white blood cells (1, 2).

The cell is easily identified by light microscopy in a blood smear by the appearance of the nucleus and coarse granules, which distinguish the appearance of eosinophils from other leukocytes (**Figure 1**). The cytoplasm contains not only the abundant and phenotypically characteristic-specific (also named secondary) granules, but also other organelles (not readily visible using light-microscopy) such as the smaller, azurophilic, and fewer primary granules. Both granules are lysosomes, storage sites for agents involved in tissue damage and inflammation. The secondary granules contain chemokines, growth factors, cytokines, and proteolytic enzymes such as the major basic protein, eosinophil peroxidase, eosinophil-derived neurotoxin, and eosinophil cationic protein (15, 23, 24, 26, 30). A predominant protein in eosinophils is galectin-10, which is now identified as a component of the peripheral cytoplasm. Upon secretion, it precipitates in tissues and body fluids as the Charcot–Leyden crystal protein, a lysophospholipase indicative of eosinophil granulocyte activity (31). The various proteases released from the specific granules contribute to the antimicrobial effect of the phagocytosing eosinophil granulocyte but may, at the same time, cause epithelial cell damage, and cytotoxicity, and contribute to fibrosis. The release of substances into tissues is damaging to microorganisms or bystander cells, causing organ damage. The concentration of proteases in body fluids may serve as a biomarker of inflammation involving eosinophils, reflecting a potential effect of circulating granule components (15, 28, 32).

Eosinophil granulocytes interact *via* their arsenal of surface-bound receptors (e.g., IgE, histamine, chemokine, cytokine, and adhesion) and the ability to secrete various proteins and other substances as mentioned above, that characterize both circulating and resident granulocytes (15, 23, 24, 33, 34). The dynamics are normally reflected in their ability to respond to infectious and inflammatory stimuli by increased numbers, eosinophilia in the blood and affected tissues, and activation. The release of preformed or stimulus-dependent chemokines, interleukins, leukotrienes, growth factors, and proteins behaves like a cascade, accompanied by a respiratory burst. The generation of reactive oxygen species upon assembly of the components of the enzyme nicotinamide adenine dinucleotide phosphate (NADPH) oxidase complex in the plasma membrane is higher per cell in eosinophils than in neutrophils (35, 36). Although the number of neutrophils is usually about 10 times higher, the increase in eosinophils in response to inflammatory or infectious stimuli contributes to the potential toxicity in the process, including harmful effects on bystander cells, through the release of granule content and reactive oxygen species. Like neutrophils, eosinophils are phagocytes that contribute to the direct control of helminth infections, and both granulocytes can form extracellular traps. This complex network of granule components and DNA is the ultimate contribution of the dying cell participating in parasitic infection and inflammation (15, 34, 37).

The phenotype of mature eosinophil granulocytes and precursor cells can be separated by the CD11b/CD62L expression, accompanied by upregulation and co-expression of various surface markers. These include C-C motif chemokine receptor 3 (CCR3, CD193), IL-5 receptor alpha (CD125), and sialic acid-binding Ig-like lectin 8 (siglec-8), a member of the CD33-related siglec subfamily, all of which are highly expressed on the surface of eosinophil granulocytes in blood and bone marrow (23, 28, 38, 39). Flow cytometry to identify eosinophils may be routinely performed, including a panel of monoclonal antibodies to detect myeloid proliferation according to Euroflow (40). However, flow cytometry may not be relevant if the eosinophil count is normal.

Being classified as an MPN, iHES carries the risk for vascular events both before and after diagnosis. The manifestations are almost always thrombotic, in both arterial and venous locations, and may be more frequently associated with clonal, primary eosinophilia. The thrombogenic potential of eosinophilia may be manifested in addition to other inherited or acquired risk factors for vascular events. The risk of bleeding may be minimal because severe thrombocytopenia or another-acquired hemorrhagic diathesis is very unusual in iHES. Retrospective analyses of HES cohorts report that 21%–24% of patients have experienced at least one event before diagnosis (41, 42). One study has linked increased expression of tissue factor (factor III or CD142), which is the initiator of thrombin generation, in eosinophil granulocytes examined in patients with iHES and secondary hypereosinophilia (43). In addition, eosinophils interact with platelets to promote atherosclerosis and thrombosis (44).

### 2.2 Epidemiology and definitions

An increase in the number of eosinophils is common and can be arbitrarily divided into three levels: mild (0.5–1.5 × 10^9^/L), moderate (≥1.5 × 10^9^/L), and severe (>5 × 10^9^/L) eosinophilia. The term hypereosinophilia may be used to characterize all cases of moderate or severe eosinophilia (3–6).

The number of eosinophil granulocytes in the blood is routinely measured by automated machine analysis as part of the differential count. Minor variations in the normal threshold, defined as 0.4–0.5 × 10^9^/L blood, may be observed between different machines and laboratories, but a manual count of (mature) eosinophils in the blood is rarely if ever, needed (45). An association within the normal range of blood eosinophil counts in adults has been reported to be correlated with several demographic factors including age, biological sex, race, BMI, and smoking status (46, 47). A diurnal variation in eosinophil count in healthy individuals has been reported to be higher at night and lower in the morning (48). Despite these variations in normal subjects, no specific recommendation has been made or deemed relevant for the interpretation of cell counts in patients with eosinophilia.

The incidence of eosinophilia varies worldwide. Although due to a plethora of reactive causes, it is more likely due to infection in tropical areas and inflammation in industrialized regions. A hospital incidence of over 10% has been reported in South Korea (49) and India (50). The incidence of eosinophilia in subjects having a blood sample taken over 10 years in the primary sector of a Western capital city was reported to be 4% in adults, reflecting that eosinophilia is a common problem to be contextualized clinically (51). In contrast, the incidence of eosinophilia in a large Canadian island district was 0.1% (52). The elevated cell count is transient in almost all patients with secondary eosinophilia, due to the impact of treatment or the self-limiting nature of the reaction, and the prevalence of eosinophilia remains low.

The definition of HES was introduced in 1968 and required that patients presented moderate or severe blood eosinophilia of unknown origin for more than 6 months, and for it to be responsible for organ damage (53, 54). The term in its original meaning is no longer applicable due to the options for treatment, the risk of irreversible symptoms, and the improvements in the diagnostic tools. Today, according to the agreed-upon definitions, HES reflects a heterogeneous group of disorders, presenting with persistent peripheral blood eosinophilia ≥1.5 × 10^9^/L on two occasions, the absence of a secondary cause of eosinophilia, and evidence of eosinophil-associated end-organ damage, justified by excessive tissue eosinophilia (55, 56).

The incidence has been reported to be 0.036/100,000 for HES in the USA (57) and 0.018/100,000 specifically diagnosed with the most prevalent primary eosinophilia, a factor interacting with PAPOLA and CPSF1-platelet-derived growth factor receptor alpha (*FIP1L1-PDGFRA*)-positive neoplasms, in France (58). The reports underline the rarity of primary eosinophilia, and more precise estimates may be difficult to collect, although the WHO ICD system provides diagnostic registry codes for different subclasses of hypereosinophilia. The *FIP1L1-PDGFRA* primary myeloid neoplasm is more prevalent in male adults (58), but otherwise, iHES and clonal primary eosinophilia are overall not gender-specific and can be diagnosed at all ages. No valid data are available on the incidence in pediatric patients (59, 60).

Eosinophilia can be classified by diagnostic tests into congenital (familial) causes as primary (intrinsic, clonal) or secondary (extrinsic, reactive) (20, 55). The diagnosis by exclusion of iHES (with symptoms due to eosinophilia) may also be clinically sub-characterized as either a myeloid (mHES) or a lymphoid (lHES) phenotype (61, 62). The lymphoid HES subtype is driven by CD3^−^CD4^+^ IL-5, producing T cells and thus secondary, non-neoplastic eosinophilia that is glucocorticoid sensitive and often associated with, e.g., angioedema, skin lesions, pruritus, and fasciitis (63). Previously, concomitant manifestations such as cardiac involvement, hepatosplenomegaly, anemia, variable leuko- and thrombocyte counts, and steroid resistance were used to phenotypically characterize a myeloid HES (61, 62, 64), similar to other MPNs. Specific diagnostic tests are required to identify patients according to the updated criteria. The revisions of the WHO classifications of malignant eosinophilic disorders by molecular diagnostic markers since 2008 have established the cluster of MLN-TK identified by specific tyrosine kinase rearrangements (3–9).

### 2.3 Symptoms of eosinophilia

The presentation of patients with eosinophilia varies considerably, in terms of symptoms and severity. Over days to months, most patients may gradually experience a worsening of symptoms related to the cause of eosinophilia, whereas symptom flares are characteristic of iHES. The presence of an increased eosinophil count in the blood may have been indolent for years, or manifestations may be due to a recent onset involving one or more organs simultaneously. The clinical context at presentation in patients with an increased blood eosinophil count is not related to the classification of primary, secondary, or iHES, because the symptoms due to eosinophilia may mimic or be involved in the diseases listed in **Supplementary Table 1** concerning organ manifestations (65–86) and **Supplementary Table 2** concerning parasitic causes (87, 88). An exception is iHE/iHE_US_, which by definition is asymptomatic eosinophilia (4–7, 20, 55).

The considerable overlap in patients presenting with eosinophilia as a clinical clue in differential diagnostics reflects how complex the correct diagnosis may be. Nevertheless, common pathologic conditions not associated with eosinophilia may manifest with similar symptoms. The recurrent question is to decide whether eosinophilia is an independent causative factor or whether the presence of eosinophilia is an additional causative factor to disorders that may have been present before the eosinophilia was noticed. Symptoms due to eosinophilia may thus be manifest in all organ systems in addition to pre-existing conditions, masked as a worsening (**Supplementary Table 1**).

B-symptoms, including weight loss, low-grade fever, and night sweats may occur in all patients with eosinophilia, whether primary, secondary, or iHES. In the individual patient, they may be attributed to cytokine signaling induced by eosinophils or other immunocompetent cells as part of a malignant, infectious, and inflammatory secondary cause (**Supplementary Tables 1**, **2**).

Reports on cohorts with eosinophilia have been published describing manifestations in iHES (65) or cross-sectional symptom registries (66, 67). Studies from Western institutions cannot be compared due to methodological differences, but the institutional reports reflect the diversity of symptoms ascribed to primary or secondary eosinophilia (**Supplementary Tables 1**, **2**). The most common organ involvement in patients with unexplained hypereosinophilia referred for examination involves dermatologic, respiratory, and gastrointestinal symptoms in approximately 45%, 35%, and 25%, respectively (65, 66). Registration of causes of unclassified eosinophilia identifies infection, allergy, and non-hematologic malignancy as common causes in different parts of the world (49, 50, 52, 61, 66, 67). These data support a multidisciplinary approach to patients who cannot be classified in a straightforward manner according to diagnostic guidelines or who do not respond adequately to proper treatment (**Supplementary Tables 1, 2**).

### 2.4 Diseases associated with eosinophilia

#### 2.4.1 Diagnostic entities

**Figure 2:**
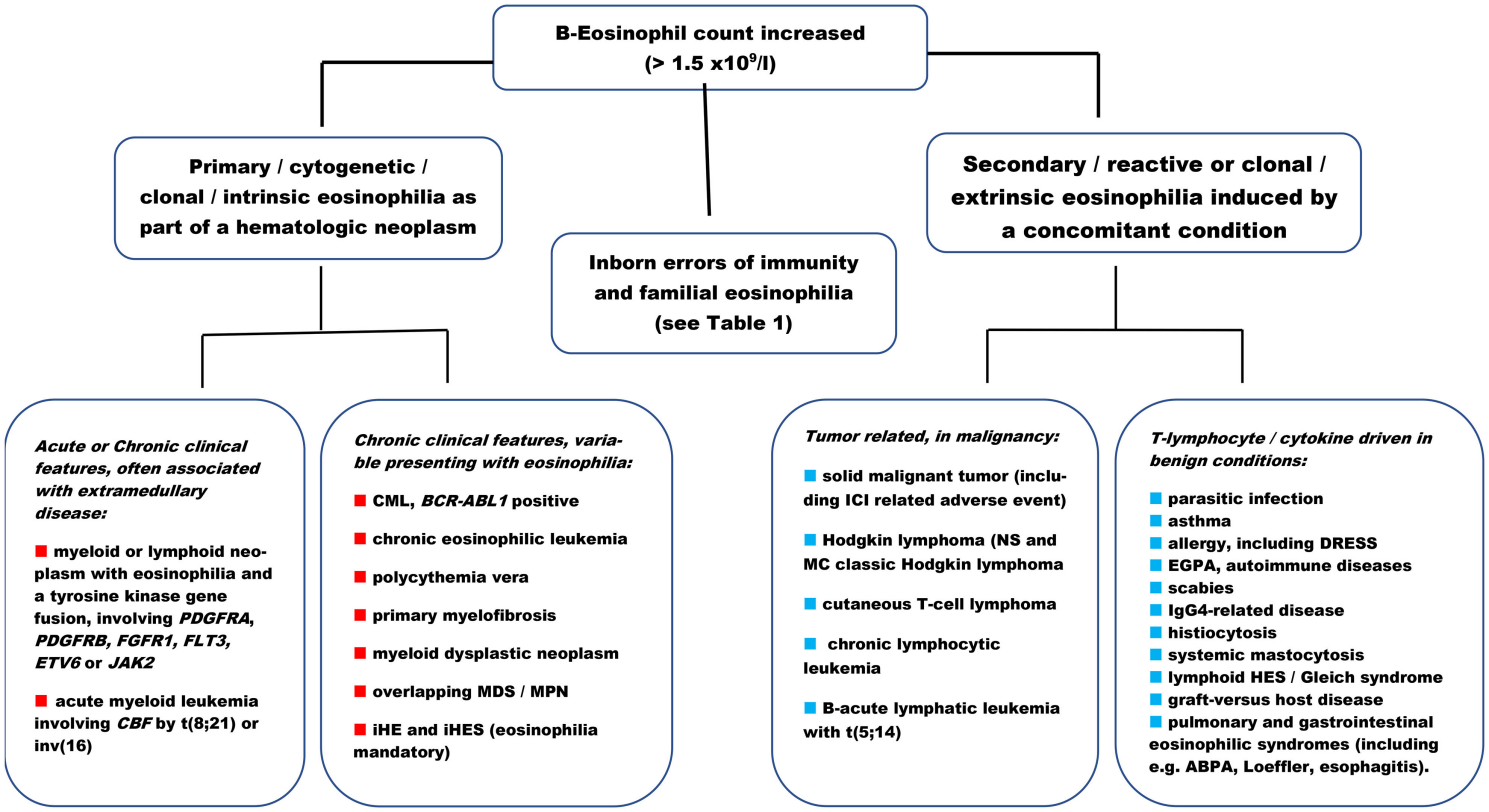
Pathophysiological algorithm and examples of diagnostic entities in patients with (moderate) eosinophilia. The diagram is not exhaustive. ABPA, allergic bronchopulmonary aspergillosis; BCR-ABL, breakpoint cluster region—abelson1; CBF, core binding factor; CML, chronic myeloid leukemia (t9;22) positive; DRESS, drug reaction eosinophilia systemic symptoms; EGPA, eosinophil granulomatous polyangiitis; ETV6, ETS variant transcription factor 6; FGFR1, fibroblast growth factor receptor 1; FLT3, Fms-related tyrosine kinase 3; HES, hypereosinophilic syndrome; ICI, immune checkpoint inhibitor; Ig, immunoglobulin; iHE, idiopathic hypereosinophilia; iHES, idiopathic hypereosinophilic syndrome; inv, inversion; JAK2, Janus kinase 2; MC, mixed cellularity; MDS, myelodysplastic neoplasm; MPN, myeloproliferative neoplasm; NS, nodular sclerosis; PDGFRA/B, platelet-derived growth factor A or B; t, translocation.

**Figure 2** depicts the clinical spectrum, contemplating diagnostic entities that approach a patient with eosinophilia in the causative context, including manifestations of organ involvement that may have overlapping presentations (**Supplementary Tables 1, 2**) (65–88). The threshold is chosen to align with the definition of iHE/iHE_US_ or iHES. Still, it applies to all cases with mild eosinophilia and patients without symptoms or with symptoms related to diagnoses other than eosinophilia. It may be important to examine in the same way patients with an increased or fluctuating eosinophil count observed over weeks to months or even years.

The descriptive manner in which eosinophilia is elucidated by pathophysiologic drivers can contribute to structuring the clinical approach and qualify diagnostic testing in a patient with newly diagnosed eosinophilia or for a second opinion at a later stage in a multidisciplinary forum. The information provided in **Figure 2** is not exhaustive, as it is not possible to present a complete list. **Figure 2** includes major groups of hematologic diagnoses as defined by the current WHO classification (9, 89). iHE and iHES are listed as clonal hematologic disorders, assuming that most cases, which at the moment are diagnosed by exclusion, may harbor a clonal driver in the eosinopoiesis (90). The algorithm indicates when eosinophils are part of the clonal disease, as described, for example, in some acute myeloid leukemias (AML) with recurrent genetic abnormalities, such as core-binding factor AML with t(8;21) or inv (16), including immature eosinophilia (91, 92). The differential diagnosis may include MLN-TK. Eosinophilia appears to be reactive and induced by cytokine signaling (IL-3) in B-acute lymphoblastic leukemia (B-ALL) with t(5;14) and with mature eosinophils circulating in the blood (93). Characterization of secondary eosinophilia can be hampered by difficulties in quantifying specific cytokine stimuli. These analyses are not done routinely at most institutions and therefore require scientific projects to accumulate data and describe the involvement of genetics as well as inflammation, e.g., in histiocytosis.

Eosinophilia is always present in iHE/iHE_US_ and iHES and is part of the diagnostic criterion. Eosinophilia is present in almost all cases of MLN-TK, caused by fusion genes involving a receptor [PDGFRA or B, fibroblast growth factor receptor (FGFR), fms like tyrosine kinase 3 (FLT3)], a transcription factor (ETV-6), or a non-receptor kinase (Janus kinase 2, JAK2). Eosinophilia is mandatory at diagnosis in chronic eosinophilic leukemia (CEL) (**Figure 2**) (6–9, 20, 58). Most patients with CML (in the chronic phase) present with mild or moderate absolute eosinophilia as part of the *BCR-ABL1* oncogene-driven leukocytosis and may rarely present as eosinophilia (94). Eosinophilia is variable, and mild, if present, in Philadelphia-negative MPNs such as polycythemia vera (PV) or primary myelofibrosis (PMF), and essential thrombocytosis (ET) but is not part of the diagnostic criteria (9, 19, 95). Overlapping MPN/myelodysplastic neoplasms (MPN/MDS) and myeloid dysplastic neoplasms are rarely associated with eosinophilia, but eosinophilia may be observed as part of the perturbed hematopoiesis (9, 17, 95).

**Figure 2** may serve as a catalog and inspiration for relevant diagnostic groups: autoimmune, infectious, clonal, etc., in the initial approach, emphasizing that the list is not exhaustive and that secondary, reactive causes are the most common (3–7, 20, 55, 64, 95) (**Supplementary Tables 1, 2**). The listed examples of benign disorders may (all) be associated with T-lymphocyte/cytokine-driven mature eosinophilia in the blood (or tissues, e.g., skin, lung, or gastrointestinal tract) and include common or very rare diagnoses such as asthma exacerbation (96), sarcoidosis (97), rheumatoid arthritis (98), and atopy (99) or IgG4-related diseases (IgG4-RD) (100). Eosinophilia may be part of the diagnostic criteria, e.g., a B-eosinophil count of at least 1 × 10^9^/L (or evidence of extravascular eosinophilic predominant inflammation on biopsy) in eosinophilic granulomatosis with polyangiitis (EGPA, formerly Churg–Strauss syndrome) (75). Similarly, a significant number of eosinophils in the sputum, airway, or blood is required to diagnose eosinophilic asthma (73).

The eosinophil count is frequently increased in parasitic infections (**Supplementary Table 2**) (87, 88, 101), allergic bronchopulmonary aspergillosis (ABPA) (88, 102), or scabies (88, 103). Eosinophilia is a predominant clinical component in lHES, characterized by marked overproduction of eosinophil factor(s) by dysregulated CD3-CD4+ T cells, which are clonal in most cases (63), and in episodic angioedema with eosinophilia (Gleich syndrome), which is a multilineage cell cycle disorder (104). The entities Eosinophilic Pulmonary Disease (EPD) (71–73) and Eosinophilic Gastrointestinal Diseases (EGID) (78) can be considered working diagnoses, including chronic, immune-mediated disorders with a multifactorial etiology and characterized by an increase in eosinophil-predominant tissue inflammation on biopsy. Organ-specific entities include several specific diagnoses such as eosinophilic esophagitis and Loeffler's syndrome, which are often accompanied by blood eosinophilia. In Loeffler’s syndrome, the eosinophilia is transient, accompanied by fluctuating, mild to severe respiratory symptoms with fever, and interstitial, migratory pulmonary infiltrates. The shading represents an accumulation of eosinophils, most often in response to parasitic infection (69–73, 78, 105) (**Supplementary Table 1**). Finally, the information provided in **Figure 2** may emphasize as clinically important that a patient may have eosinophilia for more than one reason, requiring separate treatments for proper care.

#### 2.4.2 Eosinophilic granulomatosis with polyangiitis

Eosinophilic granulomatosis with polyangiitis (EPGA) (Churg–Strauss) is a rare and potentially life-threatening systemic vasculitis. For all practical purposes, EPGA always develops in patients with pre-existing asthma (74).

The disease is characterized by predominantly small-vessel vasculitis and extravascular necrotizing granulomas associated with eosinophilic inflammation. Most patients have moderate to high blood eosinophil counts. A blood eosinophil count ≥ 1 × 10^9^/L and/or extravascular eosinophilic predominant inflammation on biopsy of affected tissue may support the current diagnostic criteria (75). It has been consistently found that 30%–40% of affected patients have antineutrophil cytoplasmic antibodies (ANCA), which can pose a diagnostic challenge in relation to the more common systemic vasculitis, although these are also rare in a tertiary rheumatology outpatient clinic (**Supplementary Table 1** and **Figure 2**).

The involvement of the lungs is the most common organ affected together with maxillary sinusitis (allergic rhinitis and/or sinus polyposis). Other major manifestations in EGPA involve the skin, peripheral nerves and kidney, as palpable purpura, mononeuritis multiplex and glomerulonephritis, respectively. Less commonly, the heart is involved with congestive heart disease symptoms and subendocardial fibrosis, and the gastrointestinal tract and the eye are affected (74, 75). Therapy includes corticosteroids (CS) and immunosuppressive agents, which overlap with the treatment of iHES.

#### 2.4.3 Histiocytosis and IgG4-related disease

Histiocytoses are very rare diseases. The prediagnostic phase is often long. It is uncommon for histiocytosis alone to present with peripheral eosinophilia, but it does occur, particularly when associated with another myeloid neoplasm. A retrospective study showed that 10% of adults with non-Langerhans cell histiocytosis have a concomitant MPN (106). Therefore, patients with histiocytosis discovered during the evaluation for eosinophilia should be offered bone marrow examination and testing for recurrently mutated myeloid genes. Hodgkinoid histiocytosis—a very rare entity—presents with eosinophilia and may mimic lymphoma (107).

In contrast to peripheral eosinophilia, tissue eosinophilia is common in histiocytosis, particularly Langerhans cell histiocytosis (formerly called eosinophilic granuloma), Erdheim–Chester disease, and ALK-positive histiocytosis (9, 95, 108–110).

In the setting of eosinophilia, the histiocytoses are most relevant as differential diagnoses to IgG4-RD, as the organ manifestations of Langerhans cell histiocytosis and Erdheim–Chester disease may resemble IgG4-RD (100, 108, 109). The distribution of lesions revealed by PET-CT scans may help to differentiate the diseases: bone involvement favors histiocytosis over IgG4-RD. Moreover, observation by imaging of perinephric changes is indicative, and demonstration of flasklike deformation in the distal femur due to meta-diaphyseal osteosclerosis, is pathognomonic for Erdheim-Chester disease. The finding of mutated v-Raf murine sarcoma viral oncogene homolog B (*BRAF*), rat sarcoma (*RAS*), or mitogen-activated protein kinase (*MAPK*) pathway genes supports a histiocytic diagnosis. Rosai–Dorfman disease is another histiocytosis that can be mistaken for IgG4-RD, as IgG4+ cells are often prominent in this condition (100).

It is currently unclear whether the eosinophilic infiltrate in histiocytic diseases is due to the disease itself or a phenomenon secondary to the inflammatory microenvironment.

IgG4-RD is an important differential diagnosis in hypereosinophilia (100). Of 100 patients with eosinophilia evaluated at a tertiary center, 9 had IgG4-RD (66). Presenting features of IgG4-RD are variable but include eosinophilia, allergy and nasal polyposis, salivary gland involvement, lymphadenopathy, sclerosing cholangitis, autoimmune pancreatitis, retroperitoneal fibrosis, and glomerulonephritis. Of note, only approximately 50% of Caucasians with IgG4-RD have elevated IgG4 levels in peripheral blood (100). Moreover, the organ manifestations of the other histiocytic diseases, Langerhans cell histiocytosis and particularly Erdheim–Chester disease may resemble IgG4-RD. PET-CT scans including the extremities to below the knee and evaluation for diabetes insipidus may help to discern between the disorders (108, 109). Kimura’s disease is a rare entity that primarily affects young to middle-aged Asians, typically causing cervical lymphadenopathy, peripheral eosinophilia, and increased IgE levels; it may resemble IgG4-RD and Rosai–Dorfman disease (111). Interestingly, Kimura’s disease has been reported to respond to an anti-Il5 monoclonal antibody (mepolizumab) (112).

#### 2.4.4 Eosinophilia in malignancies

Blood eosinophilia and/or infiltration of eosinophils in the tumor tissue is encountered in patients diagnosed with common solid tumors, and the presence of eosinophilia in blood or infiltrating solid tumors is not consistent in any neoplasm (113). A potential beneficial role may be explained by the secretion of various enzymes and cytokines by eosinophils that influence tumor immunity and reduce tumor progression (114). Severe blood eosinophilia during symptom development may be a diagnostic clue for malignancy and therefore guide the diagnostic process (115, 116). Results indicate that blood eosinophilia may be a positive prognostic factor in some malignant solid tumors, when present (113, 117), whereas the presence of blood eosinophilia after surgical resection may indicate an unfavorable prognosis, relapse, or rapid disease progression (118). The inconsistent observation of blood or tissue eosinophilia in malignant tumor entities and the lack of robust, prospective data indicate that blood eosinophilia may be used cautiously as a simple biomarker in some oncologic patients, similar to a leukemoid reaction. Nonetheless, the numerous reports and studies of eosinophilia in solid tumors add to the possible functions of eosinophil granulocytes in this developing field related to the tumor microenvironment, which awaits further clarification (119, 120).

A unique feature has emerged with the introduction of immune checkpoint inhibitors (ICIs), approved for the treatment of several cancers. Mild to moderate, though rarely severe. blood eosinophilia may be observed in less than 5% of patients, a few weeks to many months after treatment initiation. Eosinophilia may be asymptomatic, and therapy with the ICI may proceed in a small proportion of patients (121, 122). Decisions on treatment strategy are based on the individual patient’s response to targeted treatment, the severity of organ damage, and measures to control the symptoms, including CS treatment. Close monitoring of eosinophil counts, manifestations due to eosinophil activity, and response to the ICI is appropriate. Co-administration of CS to lower the eosinophil count may be acceptable, while a differential diagnosis must be excluded (**Figure 2**). Blood eosinophilia may persist after discontinuation of ICI therapy, which poses additional concerns regarding monitoring and treatment strategies for eosinophilia and malignant diseases. ICI-induced eosinophilia may have a favorable prognostic impact (122, 123). In a significant percentage of patients with ICI-induced eosinophilia, treatment must be discontinued due to eosinophil-associated organ damage, such as heart, skin, and colon (124) (**Supplementary Table 1**).

## 3 Diagnostic workup in patients with eosinophilia

The clinical challenge of identifying a diagnosis in the individual patient with eosinophilia warrants a detailed medical history and examination, in addition to the results of routine blood tests. The circumstances mirror those of patients with MPN, when serial measurements of blood cell counts show increased numbers over time, and specific analyses are often needed for clarification.

Quantification of differential white blood cell counts may be more commonly performed in specialties that treat with immunosuppressants or chemotherapy, in the interest of neutrophil granulocytes and lymphocytes. These treatments may, however, reduce the absolute number of eosinophils or be associated with fluctuating numbers, perhaps masking a concurrent condition related to eosinophilia (**Figure 2**). Observation of eosinophils in such patients at the end of cycles or in treatment-free periods may be clinically informative, providing clues for extended follow-up, e.g., in a multidisciplinary approach.

Once the presence of a repeatedly elevated eosinophil count has been confirmed, the diagnostic workup can be viewed as a stepwise process as follows:

1. exclusion of secondary causes;
2. evaluation of primary causes; and
3. diagnosis by exclusion of idiopathic hypereosinophilia.

Secondary causes are overall much more common than primary eosinophilia or iHES. A wide variety of diseases may be associated with eosinophilia, and thus a thorough medical history and clinical examination are essential to identify the causes of (secondary) eosinophilia. Relevant information includes familial predisposition, concomitant disorders, previous malignancies, medications, travel, migration and exposures, medications, and the risk of drug reactions (**Supplementary Tables 1, 2**; **Figure 2**) (65–88, 101–103, 125, 126). Since all organs may be involved in patients with eosinophilia, it is essential to ask about symptoms and observe for findings, that may not be mentioned or readily identified but may be (highly) relevant in the clinical context.

If no obvious causes of secondary eosinophilia are found, the next step is to evaluate for primary causes. This evaluation includes the following:

- complete blood cell count;
- bone marrow biopsy, aspirate, and blood for morphologic studies; and
- karyotype, molecular analysis, flow cytometry, or fluorescence *in situ* hybridization (FISH) to determine clonality.

**Figure 3:**
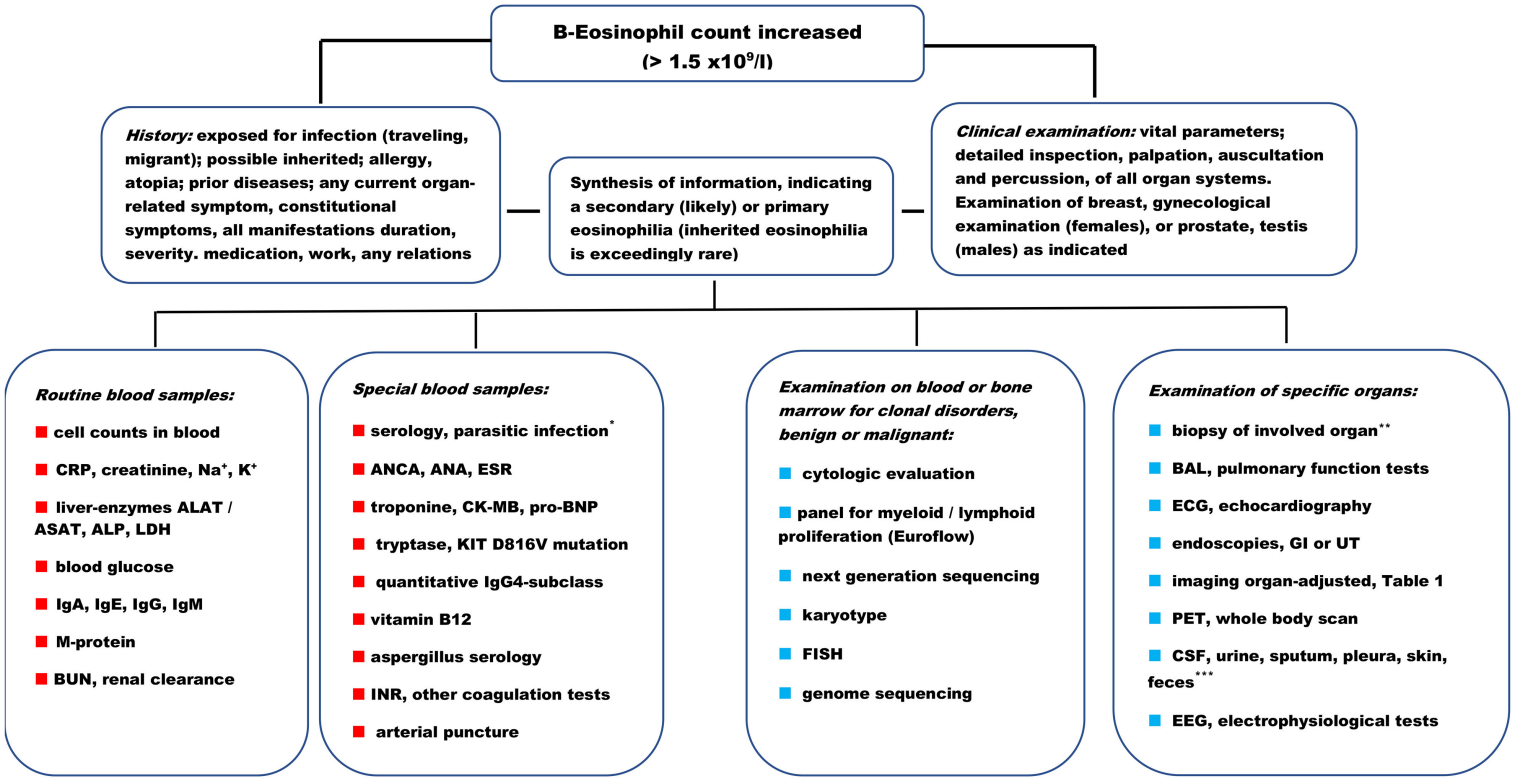
Diagnostic workup in patients with (moderate) eosinophilia. ALAT/ASAT, alanine aminotransferase/aspartate aminotransferase; ALP, alkaline phosphatase; ANA, antinuclear antibodies; ANCA, anti-neutrophil cytoplasmic antibodies; BAL, bronchoalveolar lavage; BUN, blood urea nitrogen; CK-MB, creatine kinase—myocardial band; CRP, C-reactive protein; CSF, cerebrospinal fluid; ECG, electrocardiogram; EEG, electroencephalogram; ESR, erythrocyte sedimentation rate; FISH, fluorescence *in situ* hybridization; GI, gastrointestinal tract; Ig, immunoglobulin; INR, international normalized ratio; K+, potassium; LDH, lactate dehydrogenase; M-protein, monoclonal protein; Na+, sodium; PET, positron emission tomography; pro-BNP, pro-b-type natriuretic peptide; UT, urinary tract. ⋆ See information provided in **Supplementary Table 2**. ⋆⋆ Skin, lung, lymph node, nasal polyp, liver, mucosa (GI, UT), muscle, myocardial, kidney, and brain. ⋆⋆⋆ For microscopy, culture, and other diagnostic tests (e.g., Mantoux in the skin). Proposals for blood samples and other tests to be adapted to pre-planned procedures at the department (e.g., routine laboratory packages). Proposals are not prioritized but must be selected and customized to the individual patient.

Blood counts and morphology reveal the severity of eosinophilia and abnormalities in other blood cells that may point to an underlying hematologic disease/clonal eosinophilia. Abnormalities in the morphology of eosinophils have been described in HES and CEL, but they may also be seen in reactive conditions. Bone marrow biopsy including morphology, immunophenotyping, cytogenetics, and molecular analysis may reveal an underlying hematologic disease/clonal eosinophilia. In the case of eosinophilia, FISH/cytogenetics and molecular analysis (on bone marrow aspirate or peripheral blood cells) should specifically look for *PDGFRA, PDGFRB, FGFR1, FLT3, ETV6*, and *JAK2* gene rearrangements (**Figure 3**).

The diagnosis of iHE or iHES is established when the diagnostic workup for primary and secondary eosinophilia is inconclusive.

**Figure 3** presents a flow diagram indicating primary, secondary, and idiopathic eosinophilia (3–7, 17–20, 61). The diagram is also applicable in patients with minimal eosinophilia but is aligned with iHES. The blood cell count may be repeated once or twice, if not retrospectively available over weeks and months, to ascertain a baseline value. The cell count may fluctuate and show a (significant) increase from day to day, which may be associated with worsening symptoms and the need for immediate treatment.

Examination of specific organs with biopsy or specialized analysis like magnetic resonance imaging (MRI), echocardiography, positron emission tomography (PET) scan, or organ function test (especially lung and heart) may be a part of the baseline workup for both secondary and primary causes, iHES, and iHE.

Routinely, patients with eosinophilia can be attended to in the outpatient clinic (67), but the urgency in symptom development, risk in procedures, and access to diagnostic procedures, or onset of treatment, e.g., with CS, may require hospitalization and parallel examination of primary and secondary causes. Access to specialized exams (MRI, PET echocardiography, organ function tests, etc.) may be limited to daytime or weekdays or may not be available at the current institution, which may lead to referral of patients with eosinophilia and acute manifestations to other institutions specialized in this condition.

Documentation of eosinophilic infiltration in tissues, such as skin, heart, kidney, lung, lymph nodes, and bone marrow, should be performed to demonstrate the association with iHES and primary eosinophilia or to help establish the diagnosis of a secondary course. However, an issue may be to commence cytoreductive treatment within hours of having established or recognized the potential association of eosinophilia and critical symptoms, to stabilize the patient and mitigate worsening. Response to treatment in an acute setting may be rapid—from hours to a few days. Therefore, it is valuable for the diagnostic workup if blood samples (and skin biopsy if relevant and bone marrow if possible) are obtained and kept for microscopy, flow cytometry, and clonal mutation analysis before treatment is initiated. If transfer to a tertiary center for eosinophilia is planned, a decision based on a conference with the center, or the regional hematology department is relevant to decide whether blood and bone marrow samples can be obtained for analysis before treatment starts. More invasive procedures or biopsies may be challenging to perform before initiation of treatment or transferal. Performing analysis in responding patients when the patient has a very low blood eosinophil count is unlikely to be similarly informative (**Figure 3**). The diagnostic process may be delayed, and the analysis may need to be repeated as the CS is tapered and the eosinophil count increases.

In conclusion, the diagnostic workup should be customized to the individual patient, based on organ manifestations (**Supplementary Tables 1, 2**) and interpretation of the pathophysiologic driver (**Figure 2**), and translated to conduct the relevant examinations (**Figure 3**).

In addition to the diagnostic workup, in cases of primary eosinophilia, iHE, and iHES, it may be recommended to register baseline cardiac and pulmonary function tests, notwithstanding any manifestations, but because vital organs are often involved in primary eosinophilia (3–7, 20, 41, 42, 53, 54, 58, 65–67).

## 4 Treatment of patients with eosinophilia

The first-line treatment in acute circumstances due to eosinophilia is CS, orally or intravenously at high doses (maximum 1 mg/kg prednisolone or equipotent Solu-Medrol), once a day. This is effective in approximately four out of five patients, with a gradual, often cytolytic reduction in the eosinophil count and typically an associated symptom relief. This improvement may be supported by other treatments, such as those for organ failure, infection, or a specific diagnosis, as indicated (**Supplementary Tables 1, 2**; **Figure 2**). The effect of CS on eosinophilia is rapid in CS-sensitive patients. CS can reduce the survival and function of eosinophils, block autocrine cytokine signaling, and impact the production of eosinopoietic factors derived from T lymphocytes or other immunoregulatory cells (127–130). It is not possible to predict whether a patient will respond to CS treatment (the first time CS is administered for eosinophilia) and benefit from the often-rapid effect. In patients who do not respond (sufficiently) to CS, this feature may support the interpretation of a myeloid, clonal genesis, driving the eosinophilia and overriding cytokine stimulation of eosinopoiesis, derived from T lymphocytes or macrophages. Patients with the *FIP1L1-PDGFRA* myeloid neoplasm with a tyrosine kinase fusion (**Figure 2**) are usually not sensitive to CS (58). The pattern of responsiveness to CS is often characteristic in the individual patient at the time of relapse of primary eosinophilia.

Upon diagnosis of secondary eosinophilia, treatment is initiated according to guidelines, perhaps supported by institutional recommendations, all aimed at reducing or eliminating symptoms and improving quality of life. Treatment may be directed at infectious diseases, inflammatory diseases, including autoimmune diseases, or malignancies accompanied by eosinophilia (**Figure 2**). Numerous medications are available for the management of this large patient population, which includes almost all patients with eosinophilia, and include antimicrobials, immune suppressants, anti-inflammatory drugs, or chemotherapy. Medical treatment is administered in different ways: orally, parenterally, applied on the skin, inhaled, or in other ways that target specific compartments (e.g., intrathecally), or may involve radiotherapy. CS is an example of an agent used systemically in EGPA, while it is routinely used by inhalation in asthma or ABPA. In addition, drugs supporting organ function may be needed, whether the cause is ascribed to eosinophilia or other factors. The list reflects the standard of care for all internal medicine diagnoses.

If the patient does not improve with treatment, or if (isolated) eosinophilia persists, then a follow-up assessment may be needed to exclude a concomitant disease, representing primary eosinophilia, (other) secondary eosinophilia, or very rare inborn errors of immunity or familial eosinophilia (**Supplementary Tables 1, 2**; **Figure 2**). Mild (perhaps moderate) eosinophilia may be acceptable, reflecting the number of eosinophils as a potential biomarker in patients with non-malignant disease who are otherwise, responding satisfactorily and are being monitored regularly according to standard of care (131, 132). However, an increased eosinophil count in patients with solid tumors (118) and Hodgkin’s lymphoma (133) should be followed, reflecting incomplete remission or relapse as secondary eosinophilia (**Figure 2**).

## 5 Primary and idiopathic eosinophilia

### 5.1 Specific diagnoses

The diagnostic entity “myeloid/lymphoid neoplasms with eosinophilia and tyrosine kinase gene fusions” (MLN-TK) was introduced in the 2008 WHO classification (134). The dissection of the genetic aberrations has contributed to identifying and including more patients in this clinically heterogeneous group and added information relevant to prognosis and treatment (9, 95). The neoplasms in this group are driven by constitutively active domains in tyrosine kinase fusion genes and may carry additional mutations in other genes that promote an increase in the number and survival of malignant cells (19, 135–137). Consequently, the pathophysiology of the disease and the clinical presentation of the patients with MLN-TK are very heterogeneous, emphasizing the importance of establishing a correct diagnosis. The application of cytogenetic and molecular analysis can be guided by the history (acute or chronic), examination findings (lymphadenopathy and splenomegaly), and laboratory tests (tryptase, blasts) (**Figure 3**) (138).

All the specific diagnoses included in the MLN-TK must be *BCR-ABL1* negative and instead arise due to rearrangements and abnormal gene products in *PDGFRA*, *PDGFRB, FGFR1*, *FLT3, ETV6*, or *JAK2* (9, 95, 135–137). The origin of fusion genes involves cryptic deletions or translocations of chromosomal regions during mitosis in hematopoietic stem cells, which result in dysregulated intracellular signaling and the development of distinct AML, MPN, MDS, overlapping forms, mixed phenotype acute leukemia, and T- or B-ALL or lymphoma.

**Figure 4:**
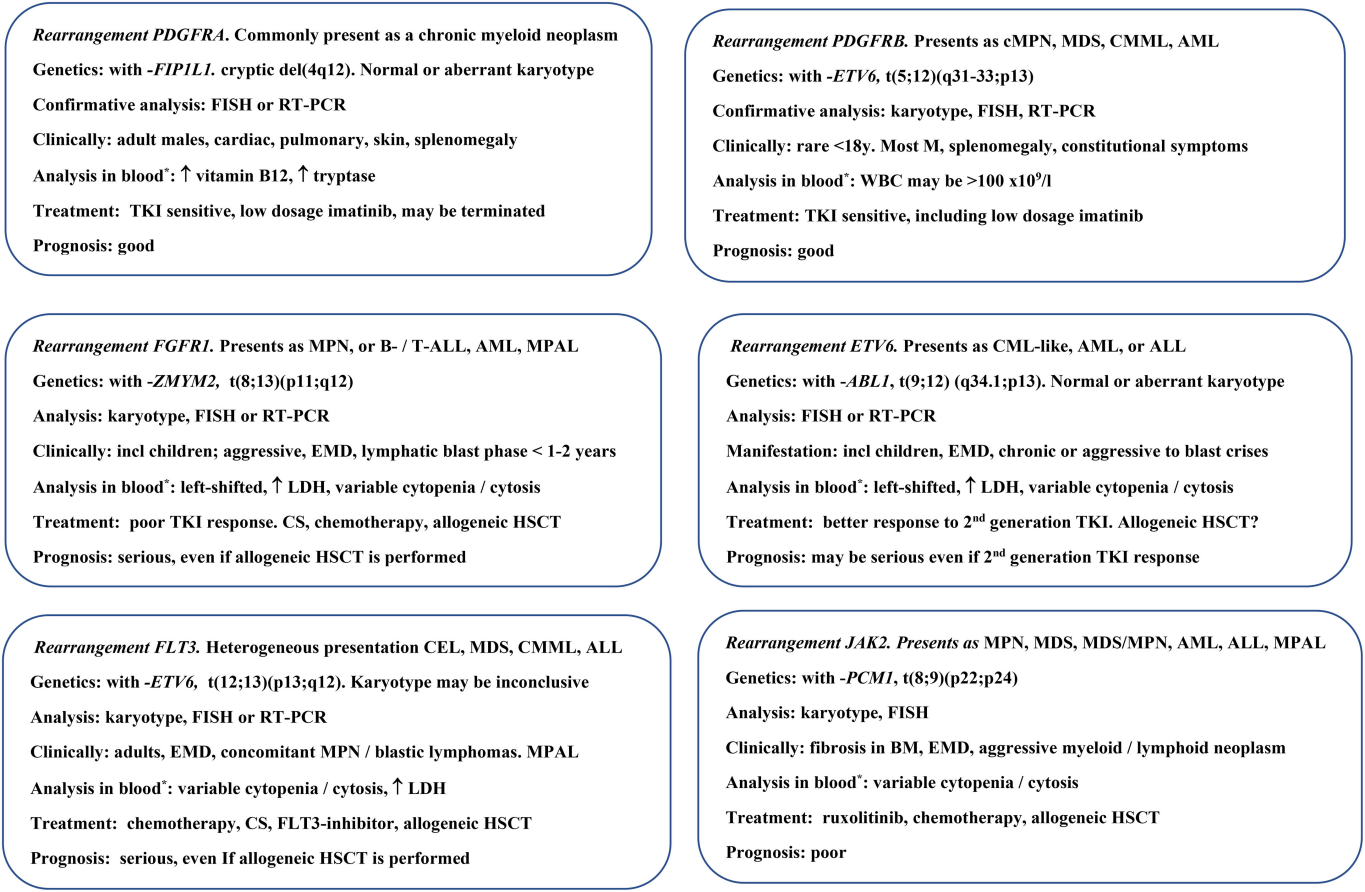
Myeloid/lymphoid neoplasms with eosinophilia and tyrosine kinase fusions (MLN-TK). One disease with a specific rearrangement occurring in the MLN-TK is described briefly. ABL1, abelson1; ALL, acute lymphocytic leukemia/lymphoblastic lymphoma; AML, acute myeloid leukemia; BM, bone marrow; CEL, chronic eosinophilic leukemia; CML, chronic myeloid leukemia; CMML, chronic myelomonocytic leukemia; MPN, myeloproliferative neoplasm; CS, corticosteroid; del, deletion; EMD, extramedullary disease; ETV6, ETS variant transcription factor 6; FGFR1, fibroblast growth factor receptor 1; FIP1L1, Factor interacting with PAPOLA and CPSF1; FISH, fluorescence *in situ* hybridization; FLT3, fms-related receptor tyrosine kinase 3; HSCT hematopoietic stem cell transplantation; incl, including; JAK2, Janus kinase 2; LDH, lactate dehydrogenase; M, male; MDS, myelodysplastic neoplasm; MPAL, mixed-phenotype acute leukemia; PCM1, Pericentriolar material 1; PDGFRA/B, platelet-derived growth factor A or B; RT-PCR, reverse transcription polymerase chain reaction; t, translocation; TKI, tyrosine kinase inhibitor, here: imatinib, dasatinib, and ponatinib; WBC, white blood cell count; ZMYM2, Zinc Finger MYM-Type Containing 2; *eosinophilia, mild–severe, almost always present.

**Figure 4** provides examples of the clinical presentation and management of MLN-TK, representing the diversity of disease in primary, neoplastic (intrinsic) eosinophilia. The figure is not exhaustive, as the individual patient may present with variable symptoms from different organ systems (**Supplementary Table 1**). The partner combinations of the fusion genes in MLN-TK influence the clinical presentation, and a more—in this context—common partner and the associated phenotype is presented as an example in each of the six categories (**Figure 4**). Specific features that may be observed in blood samples, are indicated, but are not universally observed and may be influenced by variations in disease dynamics, patient comorbidity, and latency or initiation of treatment before referral for examination, e.g., anemia, thrombocytopenia, and related symptoms. In principle, all organs may be involved in MLN-TK (**Supplementary Table 1**), and the manifestations and clinical course from the presentation may be similar to those of acute or chronic leukemia (**Figure 4**).

Eosinophilia is a hallmark of patients with MLN-TK that develops *de novo* but may be absent in a minority of patients. The increase in blood eosinophils may be mild to severe, and variable within the same MLN-TK entity. This feature reflects the complexity of the perturbed cell biology and the importance of identifying the partner gene through different analyses for clonality to confirm the correct diagnosis and possibly initiate a targeted therapy (**Figures 3**, **4**). The number of potential partners is different for each fusion gene, but one gene can be involved in different fusion gene relations, and the number of genes and related pathways involved is increasing, with at least 72 fusion gene combinations overall in MLN-TK (9, 19, 95, 135–138). A challenge in the diagnostic process is how to combine panels and methodologies with varying sensitivity and specificity in bone marrow samples (**Figures 3**, **4**). The partner in a rearrangement may not always be identified at diagnosis. Tissue samples may also be taken from enlarged lymph nodes, skin, or organs, and samples may be stored in the freezer for additional analysis if needed later.

Eosinophilia is the dominant feature of blood cell analysis in CEL and the most frequent *PDGFRA*-associated MLN-TK (9, 58, 139). The entity CEL is characterized by persistent hypereosinophilia for at least 4 weeks, organ involvement due to eosinophilia, and evidence of both clonality and abnormal bone marrow morphology (e.g., erythroid or megakaryocytic dysplasia), but does not require increased blasts (≥2% in peripheral blood or 5%–19% in bone marrow). The former CEL “not otherwise specified (NOS),” has, by this revised description, been left out of the fifth WHO classification (9, 95, 134). The result of clonal analysis in patients with CEL must exclude the other MLN-TKs specifically as primary eosinophilia, and acute or chronic MPN in general, defined by cytogenetic or mutational criteria in the WHO classification (9, 95). The information provided by cytogenetic, mutational, and cytologic analysis justifies that CEL represents an independent MPN (**Figures 2**, **4**), which often presents with organ involvement due to eosinophilia, and may share clinical features with *PDGFRA-FIPL1* MLN-TK. CEL is different from iHE or iHES, both benign conditions, and is defined by persistent hypereosinophilia in the absence of a clonal or reactive cause, by clonality, and abnormal bone marrow morphology (140). Mutational analysis for a single or concurrent clonal driver in patients with CEL has been reported in a variety of genes involved in myeloproliferation, such as *JAK2*, additional sex combs like 1 (*ASXL1*), chaperonin containing TCP1 subunit 6B (*CCT6B*), tet methylcytosine dioxygenase 2 (*TET2*), enhancer of zeste homolog 2 (*EZH2*), Casitas B-lineage lymphoma (*CBL*), or signal transducer and activator of transcription (*STAT*). The clinical phenotype may therefore be differentially associated with different genotypes (141–144). Next-generation sequencing (NGS) or polymerase chain reaction (PCR) analysis can be performed in cases without clonality by other tests to examine the differential diagnosis of CEL and iHE_US_ or iHES, which may be therapeutically important, demonstrating a potential target (**Figures 2**–**4**).

Eosinophilia may be observed in a minority of patients with MPNs such asPV, PMF, or ET. However, it may be difficult to disentangle eosinophilia as part of the neoplastic process in these cases and, alternatively, it is appropriate to consider eosinophilia in these patients to be secondary, reactive to another condition. The diagnosis of an infectious or inflammatory condition in a patient with classic Philadelphia-negative MPN is imperative for optimal management (**Supplementary Tables 1, 2**; **Figures 2**, **3**).

### 5.2 Treatment of primary and idiopathic eosinophilia

The approach may be wait-and-watch or may include any symptomatic treatment pending diagnostic clarification, such as antibiotics, xanthine oxidase inhibition, and fluid replacement, as indicated by the clinical circumstances since the dynamics and manifestations in the conditions are variable (**Supplementary Table 1**; **Figures 2**, **4**). Unless diagnostic information is available at an early stage, e.g., by flow cytometry in AML, CS may be part of a first-line treatment to reduce symptoms and the blood eosinophil count. The final treatment strategy is decided by shared decision-making with the patient, based on full information and a complete diagnostic analysis (**Figure 3**). Participation in a clinical trial is recommended for all patients with primary eosinophilia and iHES, if possible. No treatment is indicated for eosinophilia in iHES.

Imatinib is authorized by the FDA and EMA for the treatment of advanced iHES and MLN-TK with *FIP1L1-PDGFRA* rearrangement, and mepolizumab is approved as an add-on treatment for adult patients with inadequately controlled iHES without an identifiable non-hematologic secondary cause. The administration of a tyrosine kinase inhibitor (TKI) or anti-IL5 monoclonal antibody (anti-IL5 mAB) is rational given the pathophysiology of iHES and MLN-TK with *FIP1L1-PDGFRA*, which involves the potential stimulation of eosinophils by IL-5 (23–27, 145, 146) and a highly sensitive constitutively active tyrosine kinase (58, 139, 147), respectively.

The treatment strategy in all patients other than MLN-TK with *FIP1L1-PDGFRA* in the first line is therefore based on established use and extrapolation, and a highly individual assessment. This includes information on the diagnosis of CEL, type of MNL-TK or iHES, symptoms, comorbidity, and patient preferences. Adverse events or a planned pregnancy may impact the decision during treatment (3–7, 20, 61, 62, 64, 65). Treatment of primary eosinophilia and iHES includes one or more drugs to reduce and preferably normalize the eosinophil count and any additional medications indicated to mitigate symptoms caused by eosinophilia and organ symptoms. Supportive treatment is highly individualized and may involve all internal medicine specialties (**Supplementary Table 1**).

**Figure 4**; **Table 1** present the treatment options in MNL-TK, CEL, and iHES. Given the pathophysiologic mechanisms in MLN-TK, information on the clonal nature may indicate the use of TKI, chemotherapy, high-dose cytotoxic regimens, and stem cell support, including allogeneic hematopoietic stem cell transplantation (HSCT), with variable outcomes. Treatment of *FIP1L1-PDGFRA* MLN-TK shows a very high rate of durable complete remissions to low-dose imatinib, including undetectable residual disease by FISH or quantitative reverse transcriptase (RT) PCR. The potential cure of the patient is reflected by the discontinuation of imatinib in a small group of *FIP1L1-PDGFRA* MLN-TK patients (58). In contrast, the diversity of the diagnoses is reflected by indications for AML or ALL regimens in other patients of the same entity (**Figure 4**).

**Table 1:** Treatment options in chronic eosinophilic leukemia and idiopathic hypereosinophilic syndrome.

Drug	Mode-of-action in eosinophilia	Dosage (adults)	Administration	Potential adverse events	Precautions/Comments
Prednisolone (solu-medrol)	Induce lymphocyte apoptosis, reduce IL-5 production, and may induce apoptosis in eosinophil granulocytes	Starting dose: 0.5–1 mg/kg, tapering over weeks–months (40–100) daily	Oral (IV) guided by clinical circumstances Tablet (or injection)	Osteoporosis, diabetes mellitus, anti-pyretic, risk for infections, increase in serum cholesterol and triglycerides, hypertension, reduced wound healing, impaired skin integrity, mental disturbance	Check blood-glucose and lipids, institute osteoporosis prophylaxis and BMD monitoring if other risk factors and/or repeated courses or maintenance treatment with CS is administered. Slow tapering in weeks to avoid adrenal insufficiency after long-term treatment. Patients may carry an information card
Interferon-alpha 2a	Receptor binding and immunomodulation, acting on lymphoid and myeloid cells	62.5–180 μg 7–10 (sometimes 14) day interval	SC (preceded by paracetamol). Pre-filled syringes	Mental and physical fatigue, gastrointestinal myalgia and arthralgia, anemia, thrombopenia, and/or leukopenia, hepatotoxicity, and thyroid dysfunction	Recommend gradually increasing dosage over weeks, according to vial content, to increase tolerance. Acceptable in pregnancy. To be self-administered by the patient (or caretaker)
Hydroxyurea	Cytoreductive, phase-related inhibition of DNA synthesis	500–1,500 mg once daily (rarely less, not recommend a higher dose)	Oral Tablet or capsule, one dosage size	Pan-cytopenia, an increase in MCV, oral mucositis, alopecia, skin ulcers, melanoma, and possibly carcinogenic	Avoid exposure to sunlight. Later-line treatment in patients <60–65 years due to long-term risk. Consider sperm deposit. Contraindicated in pregnancy, lactation
Mycophenolat mofetil	Inosine-5’-monophosphate dehydrogenase blocking agent, inhibiting T- and B-lymphocyte proliferation and function, immune suppressive	500–1,500 mg in one or two dosages daily	Oral Tablet	Abdominal pain, anemia, thrombo- and/or leuko/lymphopenia, infection, hepatotoxicity, alopecia	Recommend increasing dosage gradually and await an effect in at least 3 months. Contraindicated in pregnancy, lactation
Azatioprin	Antagonist of purine metabolism, immune suppressive	50–150 mg once daily	Oral Tablet, variable dose	Anemia, thrombo- and/or leuko/lymphopenia, hepatotoxicity, infection	May be used in pregnancy, but not while lactating. Interactions
Cyclosporine A	Immune suppressive, inhibition of the production of cytokines, e.g., IL-2, involved in T-lymphocyte activation	100–200 mg once daily (low dosage)	Oral Capsule or mixture	Tremor and cramps, hypertension, renal insufficiency (but less than observed in organ-transplants)	Contraindicated in renal insufficiency. Monitoring P-concentrations should not be needed
Methotrexate	Immune suppressive, inhibits purine and pyrimidine synthesis, reducing T-lymphocyte cytokine signaling	5–20 mg once weekly	Oral Tablet, variable dose	Anemia, thrombo- or leuko/lymphopenia, infection (including HZV re-activation), stomatitis, gastrointestinal, and pneumonitis	Contraindicated in pregnancy and lactation. Regular monitoring of blood cell-counts. HZV vaccination?
Mepolizumab	Anti-IL5 monoclonal antibody	300 mg every 4 weeks	SC	Abdominal and back pain, eczema, and allergic reactions.	Risk for parasitic infections (**Supplementary Table 2**)
Imatinib	Tyrosine kinase inhibitor	100–400 mg daily	Oral Tablet, variable dose	Muscle cramps and stiffness, abdominal pain, periorbital or extremity edema, anemia, thrombo- and/or leukopenia, alopecia	Dose relation in adverse events. Contraindicated in pregnancy and lactation

BMD, bone mineral density; HZV, herpes zoster virus; IL-2, interleukin 2; IL-5, interleukin 5; IV, intravenous; SC, subcutaneous.

The treatment of CEL and iHES reflects the possible lymphoid or myeloid nature since the specific mechanism of the disease is not known in iHES and may not be translated from a clonal finding to a therapeutic agent in CEL (**Table 2**). There are no randomized clinical trials (RCTs) or robust data to support international agreement on the first or subsequent lines of treatment, after CS, and the decision will depend on the evaluation of the individual patient and treatment options.

**Table 2:** Ongoing clinical trials in patients with primary eosinophilia.

Drug (NCT number)	Title	Start date–estimated completion date	Estimated enrollment (age at inclusion)	Phase (Design)	Comparator	Primary outcome (route of administration)	Locations (number of sites)
Benralizumab (anti-IL-5r mAb) (NCT04191304)	A phase 3 study to evaluate the efficacy and safety of benralizumab in patients with hypereosinophilic syndrome (HES) (NATRON)	22 July 2020–4 November 2024	120 participants (≥12 years)	Phase 3 (Double-Blind)	Placebo	Time to first HES worsening/flares (SC)	United States, Austria, Belgium, Denmark, France, Germany, Israel, Italy, Japan, the Netherlands, Poland, Switzerland (46)
Depemokimab (anti-IL-5 mAb) (NCT05334368)	Depemokimab in participants with hypereosinophilic syndrome, efficacy, and safety trial (DESTINY)	6 September 2022–30 May 2025	120 participants (≥18 years)	Phase 3 (Double-blind)	Placebo	Frequency of HES flares (SC)	United States, China, Japan, Republic of Korea, Spain (12)
Imatinib (TKI) Ruxolitinib (JAK inhibitor) (NCT00044304)	Tyrosine kinase inhibition to treat myeloid hypereosinophilic syndrome	26 September 2002–1 January 2026	60 participants (>2 years of age for imatinib, and ≥18 years of age for ruxolitinib)	Phase 2 (Open-label)	None	Peripheral blood absolute eosinophil count (oral)	United States (1).
Ruxolitinib (JAK inhibitor) (NCT03801434)	Ruxolitinib in treating patients with hypereosinophilic syndrome or primary eosinophilic disorders	15 November 2019–21 October 2023	25 participants (≥18 years)	Phase 2 (Open-label)	None	Overall response rate (oral)	United States (4)
Mepolizumab (anti-IL-5 mAb) (NCT04965636)	Study in pediatrics with hypereosinophilic syndrome (SPHERE)	14 July 2022–13 September 2023	25 participants (6–17 years)	Phase 3 (Open-label)	None	Number of HES flares experienced by participants per year (SC)	United States, Argentina, Spain (9)
Benralizumab (anti-IL-5r mAb) (NCT02130882)	Study to evaluate safety and efficacy of benralizumab in subjects with hypereosinophilic syndrome (HESIL5R)	19 May 2014–31 December 2023	20 participants (≥18–75 years)	Phase 2 + 3 (Double-blind)	Placebo	Number of participants with a 50% reduction in peripheral blood eosinophilia at week 12 (SC)	United States (1)

anti-IL-5, anti-interleukin 5; anti-IL-5r, anti-interleukin 5 receptor; HES, hypereosinophilic syndrome; JAK, Janus kinase; mAB, monoclonal antibody; SC, subcutaneous injection; TKI, tyrosine kinase inhibitor. Database search of clin.trial.gov March 2023.

CS is the drug of choice when immediate treatment is indicated in CEL and iHES, until clarification. Maintenance treatment, if CS is effective, should include steroid-sparing agents, due to the chronic nature or repeated need for CS, while tapering CS. Lack of CS sensitivity may indicate a “myeloid” phenotype, supporting, for example, interferon-alpha or hydroxyurea as a non-targeting treatment as the next line of treatment. Access to serum IL-5 or other cytokine levels may guide treatment with an immunosuppressive drug. However, validated and robust prospective results are not available, and pretreatment with CS may impact the analysis (145). The decision to introduce methotrexate, azathioprine, cyclosporine, or mycophenolate, instead of, for example, interferon-alpha or hydroxyurea, remains a clinical one (**Table 1**). The administration of these drugs is supported by case reports, small studies, retrospective analyses, and established use extrapolating from experience in lymphoproliferative and myeloproliferative diseases (63, 104, 148–151) (**Table 2**).

The lowest dose of CS or any agent should be used, and combination treatment may be administered, as in selected cases with PV or PMF, to reduce adverse events and maintain optimal efficacy. Efficacy may last from weeks to 2 - 3 months, preferably introducing only one drug at a time. Prophylaxis against osteoporosis should be initiated at an early stage in CS-sensitive patients with risk factors for bone density reduction, as CS may be used for months and repeatedly. Prophylactic antimicrobials for *Pseudomonas* infection may be contemplated in selected patients with chronic lung disease and long-term therapy with immunosuppressive agents (**Table 1**).

Imatinib is approved for advanced iHES, which is a heterogeneous group clinically identified with persistent symptoms due to eosinophilia, and unresponsive to administered lines of treatment. Imatinib may be effective in CEL or iHES because fusion genes, not detected by analysis, may be sensitive to TKI (152). A standard dose may be started and tapered if effective to the lowest effective dosage. Response to any treatment in CEL or iHES is variable (**Table 1**).

There are no internationally agreed-upon definitions of response to treatment in primary eosinophilia or iHES. Reduction in symptoms, flares, eosinophil count (complete response requires durable normalization), and any biomarker, particularly the clonal marker, may reflect a satisfactory effect.

Irrespective of the choice of treatment in CEL or iHES, drug discontinuation may be planned after months to years of treatment. It may be reasonable to taper the dosage, considering the drug and being aware that relapse of symptoms, typically manifested at the time of diagnosis is a risk. Patients with rare diagnoses such as primary eosinophilia or iHES should continue to be monitored and treated in specialized departments.

## 6 Ongoing research in primary eosinophilia

Several clinical trials have been completed or are ongoing with anti-IL5 biologicals in patients with secondary eosinophilia, such as eosinophilic asthma (153), eosinophilic esophagitis (154), and EGPA (155). The results of two RCTs have been published (145, 146) and several clinical studies are ongoing in targeted therapies regarding iHES and clonal eosinophilia. The rarity of these patients is a concern in the planning of clinical studies, and most centers may only be able to enroll one or two patients in a trial. This is a burden for the individual (hematology) department and may be a reason to decline participation. Referral to centers may be a way to mitigate this challenge, but the administrative work and bureaucracy are a challenge for the conduct of clinical studies, which may impact research activity in primary and idiopathic eosinophilia (156).

**Table 2** provides information on six trials currently ongoing in Western countries, some of which are still recruiting. The table reflects the challenge due to the rarity of the diseases to conduct active comparator RCTs, instead of “physician’s choice,” which, in primary eosinophilia and iHES, may be very heterogeneous (**Table 1** and **Figure 4**). This may impact the interpretation of the outcome and the option for blinding the study. Alternatively, blinding of the randomized trial is possible and valuable in placebo-controlled designs, which isolate the effect of the experimental treatment even as an add-on to a standard of care, e.g., CS and hydroxyurea. Inclusion criteria are defined across all ages, reflecting the age distribution of patients with this diagnostic entity. The trials in pediatric patients are of particular interest in order to mitigate any long-term effects of alternative immunosuppressive, immunomodulating, or cytoreductive treatments. It is also an added value for adult patients with iHES or primary eosinophilia to offer biological therapy to achieve disease control by targeted agents with acceptable safety profiles, possibly lifelong, as in all MPNs.

The primary outcomes of the trials are listed and reflect the interest in restricting eosinophilia (by blood cell count) or symptoms (by the rate of response or flares), which are rational and objective endpoints and relevant to patients to reflect a treatment benefit. In this context, the information presented in **Table 2** shows that the administration of all agents in the current trials is either orally or subcutaneously, which is beneficial for chronic treatments and allows home administration. If possible, novel drugs in clinical trials for primary eosinophilia or iHES administered intravenously should be avoided in order to reduce patients’ time consumption/travel and stay at the hospital during treatment, and to reduce the use of resources at the department. This pattern, reflected in **Table 2**, is endorsed.

Dexpramipexole is an orally bioavailable synthetic aminobenzothiazole that has demonstrated encouraging results when treating HES with this potentially steroid-sparing agent (157, 158). One trial was started in 2014, but the status of the open-label, phase 2 study to evaluate the safety and efficacy of dexpramipexole (KNS-760704) in adults with iHES is unknown (NCT02101138).

Clinical studies of a second-generation TKI targeting *BCR-ABL1*, like dasatinib and nilotinib in the second line in patients with *FIP1L1-PDGFRA* MLN-TK, failing imatinib, or a *JAK2*-targeting TKI, like ruxolitinib, have possibly met the challenges of conducting clinical studies, even single-arm trials, in rare populations. One consequence may be the off-label use of these drugs, but it is pivotal to accumulate data on their efficacy and safety in patients with MLN-TK. This may be supported by centralized treatment of the patients or reporting to international registries.

## 7 Quality of life in primary and idiopathic eosinophilia

Patient-reported outcome (PRO) measures are today recognized as key endpoints in all clinical trials within hematological disorders (159). For *BCR-ABL1*-negative MPN, disease-specific tools for quality of life (QoL) assessment have been developed, e.g., the Myeloproliferative Neoplasm (MPN) Symptom Assessment Form Total Symptom Score (MPN-SAF-TSS) in ET, PV, and PMF (160), and in myelofibrosis specifically (161). The symptom burden among patients with MPN is highly correlated with QoL (162).

However, no specific tool to capture PRO in iHES, MLN-TK, or CEL has been developed and validated, and the MPN-SAF-TSS did not when established, include patients with these rarer acute and chronic myeloid conditions. Currently, general questionnaires like the European Organization for the Research and Treatment of Cancer Quality of Life Questionnaire (EORTC QLQ-C30) (163, 164), the simpler EuroQol 5-dimensional questionnaire (EQ-5D-5L) (165), or the Patient Reported Outcome Measurement Information System (PROMIS) Short Form (SF) (166) are implemented and recommended in the monitoring of patients and clinical trials in oncology (166, 167). The EORTC-QLQ-C30 in particular has the advantage of being available in more than 100 languages and having electronic records, which may be useful in clinical trials. These tools may be feasible for capturing relevant subjective issues in patients with iHES or primary eosinophilia. The data on QoL are registered as secondary or exploratory endpoints and may therefore be of limited value in single-arm studies or otherwise unblinded trials due to the risk of bias. The multi-faceted manifestations of the target population (**Supplementary Table 1**) pose a challenge when trying to develop one homogeneous questionnaire in clinical trials of idiopathic and primary eosinophilia (**Figures 2**, **4**). The placebo-controlled, blinded RCT of mepolizumab in iHES included QoL registered by SF12 (145). The survey demonstrated that baseline parameters were similar in the experimental and control groups and that QoL did not deteriorate during the study period. QoL measures are included in many ongoing trials (**Table 2**). Robust studies of QoL in primary eosinophilia, iHE, and iHES outside a clinical trial have not been done.

Tools like the Patient Global Impression of Change Item (PGIC) or the Patient Global Impression of Severity Item (PGIS) may be acceptable in clinical studies and for population studies due to the lack of more specific measures (168, 169). The Physician Global Assessment (PGA) has been used in several clinical settings as a disease activity instrument on a visual analog scale and was originally developed for systemic lupus erythematosus (170). However, PGA may not be adequate from an overall perspective in iHES or clonal eosinophilia because, although some symptoms may be objective, several are not (**Supplementary Table 1**), and the PGA may also be biased, influenced by the physician’s experience and circumstances of evaluation.

Thus, today, information regarding QoL in iHES and clonal eosinophilia outside clinical trials should be captured and monitored by detailed interviews with the individual patient due to the known plethora of symptoms in the population. This is a time-consuming challenge in the clinic, and electronic tools for comparing symptoms and variables, e.g., cell counts in blood, may be an important tool to improve the inclusion of this vital information when monitoring patients in the future (171).

## 8 Multidisciplinary team in eosinophilia

### 8.1 Description of the collaboration

A multidisciplinary team (MDT) in a hospital is a function specifically suited to cases with complex care needs due to the variable symptoms, diagnostic challenges, and individualized treatment. MDTs are appropriate for rare diseases because expertise is required to provide optimal care and coordinating work in professional MDTs also promotes improvement in patient management. This has been well described in the management of patients with benign or malignant surgical and medical disorders and in research (172–177). MDTs may also be called tertiary centers.

Establishing an MDT has to take into account the different facilities and disciplines at hand, and the effort necessary in an MDT requires a number of clinical and diagnostic facilities to join the collaboration. MDTs are most often organized in regional or university hospitals with centralized functions in several specialties, and thus with extensive and recurrent interaction with local/regional hospitals based on collaborating policies. This development is in line with the centralization of certain functions, to aim for and maintain the highest degree of competence. MDTs and centralized functions reflect the same interest but differ in—at least—the fact that centralized functions are often allocated to one department based on daily routines, whereas MDTs are only operational when several diagnostic and clinical departments have established and agreed to a manner in which to collaborate, as a local policy. The centralized function is expected to run 24/7, whereas MDTs typically have planned meetings at weekly intervals.

A tertiary center for eosinophilia is a specialized function serving a large population since the number of patients with a primary or idiopathic cause in need of specialized treatment at the department of hematology and the incidence of complex secondary cases is low. Inborn errors of immunity and familial cases are exceedingly rare (**Figure 2**). The population of Denmark is 5.85 million (in 2021), and two MDTs were allocated by government decision in 2017 at two university hospitals, each in a different part of the country. One is in the capital region (at Rigshospitalet, in Eastern Denmark) and the other is in the Southern Denmark region (at Odense University Hospital). Both MDTs are open to referrals from all five regions of the country, primarily for adult patients. Individual patients with eosinophilia can decide which MDT is more convenient for them to attend, but the contact with local/regional departments can be continuous due to the collaboration between departments at different institutions. Similarly, tertiary centers for other rare diagnoses have been established at Danish university hospitals. The Center for Eosinophilia is physically located in the Department of Hematology and collaborates with the six other hematology departments in Denmark, which are routinely involved in the management of patients following an initial referral at a regional level. The Department of Hematology may be appropriate to host the MDT due to the special differential diagnosis, including idiopathic and primary eosinophilia, and also including secondary cases of histiocytosis, malignant lymphoma, and leukemia, whereas the majority of secondary eosinophilia cases are optimally treated at other specialized departments (**Supplementary Tables 1, 2**; **Figures 2**–**4**).

### 8.2 Elements and benefits of an eosinophilia MDT

**Figure 5:**
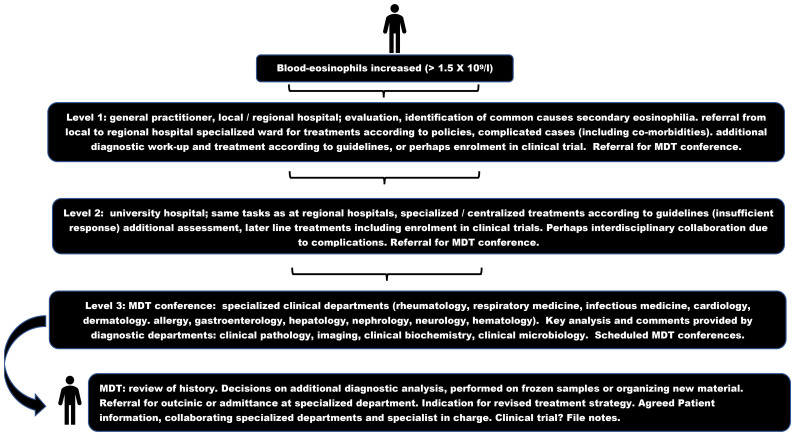
Multidisciplinary team flowchart in eosinophilia. MDT multidisciplinary team.

**Figure 5** shows the principles of patient flow and functions in an eosinophilia MDT. It is important that GPs are also aware of the tertiary centers, e.g., through the dissemination of knowledge at regional meetings and in national medical journals (67, 178) (level 1) and that the specialized departments at the MDT hospital all serve as a filter for a second opinion in the MDT (level 2). Furthermore, GPs should be aware that there are no formal criteria for listing a case for an upcoming conference, which is planned regularly every 4–6 weeks. The MDT program can be planned 6 months in advance, each meeting lasting 60–75 min. The upcoming conference is announced *via* a mailing list to the colleagues in the MDT network and is sent out in advance with anonymized information on usually three to five patients. More detailed information can be retrieved in the secure areas of the hospital’s electronic filing system, which can be shared with all other hospitals in the region. The email information includes a video link for virtual participation in the MDT, which has been a positive outcome of the COVID pandemic (179). In this way, videoconferencing facilitates participation. The two centers for eosinophilia in Denmark organize conferences together four times a year to disseminate knowledge. This activity is made possible due to the use of virtual rooms. In principle, these conferences are open to all interested colleagues (nationwide) and participation is optional. The schedule for the MDT can include fixed days or be a rolling sequence to accommodate the numerous other activities on the ward, in the outpatient clinic, and at other conferences.

A major benefit of the organization of an MDT in eosinophilia is that it allows for direct contact with colleagues participating in the MDT during normal working hours. Colleagues can be contacted *via* an MDT telephone list and are more familiar with the task, and thus are more likely to be able to provide a qualified opinion directly than can be expected from both junior and senior doctors who are generally on-call. Naturally, cases can be discussed in a department within the MDT network, between on-call colleagues and colleagues participating in an MDT, if this is more feasible. It is our experience that an option for personal contact, through telephone lists for the MDT, is one of the aspects facilitating the process when, for various reasons (clinical circumstances, time, and presence) a discussion cannot await the next scheduled MDT to make decisions.

**Figure 5** illustrates that most patients are referred from highly specialized departments at the MDT hospital or from collaborating hematology departments (67). The individual patient is presented by the physician most familiar with the case (most often the treating specialist). The case presentation may be in PowerPoint format, which can support the educational dimension of MDT conferences. Patients can be discussed in the MDT either during admission to the hospital or during follow-up in the outpatient clinic, reflecting the wide range of symptoms and severity in the target population (**Supplementary Tables 1, 2**; **Figures 2**–**4**). The purpose of discussing an eosinophilia case in the MDT is to agree on a second opinion, including the need for additional diagnostic tests, e.g., due to persistent eosinophilia and symptoms despite relevant treatment for secondary eosinophilia, and also to establish diagnoses of iHE and iHES *per exclusionem*. The next step is a treatment decision, according to the working diagnosis, to be implemented at the appropriate specialized department, either in the outpatient clinic or during admission to the ward. The scheduled conferences, both in the individual center and in the two national centers, must always include a plan for informing the patient (usually by phone) and any department or function involved (either by verbal information or by referral in writing). All decisions should be recorded as a conference note, and the next MDT will provide the opportunity for follow-up (**Figure 5**).

## 9 Discussion

A patient-centered function is fit for purpose due to the complex and challenging manifestations, diagnostics, and options for targeted or revised therapeutic strategy in a small proportion of adult patients with eosinophilia. This is the first presentation of the rationale for the MDT in eosinophilia (**Supplementary Tables 1, 2**; **Table 1** and **Figures 2**–**4**). The MDT function is a well-established tool across medical disciplines (172–177), and MDTs have been implemented in departments treating eosinophilic esophagitis, possibly improving patient outcomes (180, 181), and have also been considered in Loeffler’s endocarditis (182). However, one difference between the MDT for patients with eosinophilia and other scheduled MDTs is that the MDT for eosinophilia typically starts by challenging and discussing the working diagnosis (**Figure 5**), whereas the diagnosis has been established unquestionably in (almost all referred patients) MDTs. The number of clinical and diagnostic departments in an eosinophilia MDT is high, requiring input from all medical specialties and diagnostic expertise (**Figure 5**). MDTs for established diagnoses in one specialty include additional expertise in specific fields, such as radiotherapists, physiotherapists, specially trained nurses, and nutritionists.

The MDT conferences in eosinophilia, if executed virtually, require an optimal technology for sound and image quality (179), and the sharing of specific parts of the patient’s files among the MDT participants. These may include data from blood samples, medical history and clinical findings, CT-PET-MRI scans and descriptions, and light microscopy images of tissue samples. It is very important to prioritize the patient information and decide on the physician in charge of the next steps, and the procedures. Activities must take precautions to avoid disclosure of personal data when disseminating information by mail or virtual systems.

The MDT forum can facilitate educational activities, sharing of experiences at all levels of medical training and specialization. Moreover, the collaboration between the two MDTs in Denmark supports the possibility of participating in international clinical trials, e.g., in iHES (**Table 2**), because the potential number of patients to be included is higher than in the individual centers. The accumulation and interest of patients with eosinophilia in MDTs facilitate research, e.g., cohort studies in chronic lymphocytic leukemia demonstrating the impact of eosinophilia at diagnosis (183) and a retrospective study advocating PCR analysis for T-cell receptor status in the diagnostic workup (**Figures 2**, **3**), to be performed only if clonality has been demonstrated by flow analysis of lymphocyte subpopulations in blood or bone marrow samples (67).

The differential count in blood and readily identifiable eosinophil granulocytes in blood smear (**Figure 1**) are a simple source of information to guide toward the broad spectrum of conditions associated with eosinophilia, as opposed to conditions characterized by isolated neutrophilia, monocytosis, or lymphocytosis (1, 2). While isolated blood eosinophilia is characteristic of iHE, concomitant aberrations in other cell lines indicate other hematologic conditions, including iHES and MPNs, in addition to eosinophilia due to secondary causes (**Figure 2**). The biology of eosinophil granulocytes is well described (21–44), as is their role in various pathophysiologic states (**Supplementary Tables 1, 2**; **Figure 2**). The narration of eosinophil functions expanded from “a phagocyte” in innate immunity to a potential key player in the orchestration of homeostasis, normal development, and remodeling (15, 26, 28, 38). However, results from genetic knock-out models in animals, and more recently the possibility to observe any impact of treatments that deplete circulating eosinophils by IL-5 and IL-5 receptor-targeting treatments in functional knock-out humans, challenge the notion of the precise role of eosinophils in health (184–187). More robust results in patients treated with anti-IL5 biologicals or other targeted treatments, in primary or secondary eosinophilia, lowering the eosinophil count durably and maximally as well as research in this field is warranted.

Eosinophils are distributed after extravasation in numerous tissues, but it remains to be understood in more detail what triggers eosinophils to cause various end-organ manifestations in the individual patient (**Supplementary Table 1**). The distribution of eosinophils in human tissues is normally organ dependent and varies considerably. The distribution may be explained by tissue-specific chemokines or reflect the existence of eosinophil subpopulations *in vivo* (26, 29, 188, 189). The impact of cell subpopulations in primary or idiopathic eosinophilia has not been studied but may contribute to explaining the manifestations in different organs (65).

Current isotope scintigraphy is not readily applicable to eosinophils, in contrast to the information obtained from tracing neutrophils with a clinical benefit in diagnosis and treatment (190). An eosinophil-specific tracing technique could be a major step forward in the diagnosis and management of patients with eosinophilia, replacing or supporting invasive procedures. Perhaps monoclonal antibodies targeting epitopes, preferentially present on eosinophils, such as the IL5-receptor (**Table 2**) or siglec-8, could be labeled with an isotope serving as a tracer. However, cell death upon binding to the eosinophil plasma membrane would (most likely) be rapidly induced (191, 192). It may be considered whether antibody-functionalized magnetic particle imaging tracers could be a technique applicable to eosinophil granulocytes (193). Currently, imaging techniques indicating inflammation, like PET or gadolinium MRI and biopsies, remain part of the gold standard to demonstrate the involvement of eosinophils in damage.

The natural history of iHE and iHES, including triggering events for progression, is not well understood. Data on the population are available in retrospective, descriptive studies, which indicate the presence of severe anemia, hepatosplenomegaly, older age, and cardiac involvement in a scoring system to be prognostic. Patients with a low-risk score have a 5-year survival of over 95%, while a high-risk score indicates a 5-year survival of 62% (20, 194). Prospective MDT-orchestrated studies and multicenter studies can be a tool to accumulate data, as in the ongoing French COHESION study started in 2019 (NCT04018118). Similarly, the U.S. longitudinal study of familial hypereosinophilia started in 2005 (NCT00091871), may clarify details of the natural history of these rare disorders.

The number of eosinophils in the blood is not itself well-correlated with manifestations in patients with iHES (194, 195). Data have been presented that end-organ damage, to a variable extent in heart, lung, skin, and other tissues, may be explained by extravasation of eosinophils during the process, which affects the eosinophil count, and the risk of symptoms due to eosinophilia does not increase proportionately with counts higher than 1 × 10^9^/L. The clinical implication is that a normal level or even a low number of eosinophilic granulocytes in circulation is not a safeguard for excluding the risk for eosinophilic involvement in symptomatic patients (196, 197). The clinical implication is that the number of eosinophils in the blood may not in itself be a risk factor in hypereosinophilia, and the clinical assessment and indication for treatment in asymptomatic patients require careful patient information to react to symptoms. The clinical explanation for a correlation between symptoms and eosinophil count, when treatment is started, may therefore reflect the effect of lowering an increased eosinophil count and the clearance of eosinophils in the affected tissues.

Finally, the prognostic information of the blood eosinophil count on admission to the emergency department has been studied in a large, unselected, adult population. Moderate or severe eosinophilia is a risk factor for a significantly increased 3-month mortality, in patients older than 70 years with hematologic disease and an elevated C-reactive protein (198). The results emphasize that awareness of the eosinophil count is a diagnostic clue in the management of hypereosinophilic patients to reduce diagnostic delay, especially of the underlying malignancy (8, 198) (**Figure 2**).

The iHE_US_ and iHES, respectively, may be examined in more detail at diagnosis to improve the classification. Whole exome sequencing and genome-wide methylation analysis identifying novel disease-associated mutations and methylation patterns, may be appropriate tools to convert the idiopathic status into identifiable mutation-driven entities (90, 141–144). A proportion of cases may thus be contextualized within the MLN-TK or CEL groups, and perhaps be of therapeutic relevance. Casuistic reports of MPN or MDS presenting as iHES, reflecting a diagnostic overlap may not represent a pathophysiologic continuum (199). Rather, the reports reflect the complexity of establishing a strict classification in the chronic myeloid neoplasms with eosinophilia.

The introduction of IL-5 targeting treatment in neoplastic or idiopathic hypereosinophilia mirrors the increasing interest in recent years in mABs targeting the IL-5 pathway for the treatment of hypereosinophilic conditions such as severe asthma and EGPA (33, 153, 200). **Table 2** shows that trials are ongoing in two mABs against free IL-5 with mepolizumab or a long-acting formulation (depemokimab), both of which neutralize IL-5 and prevent receptor binding. One study involves an mAB targeting IL-5r (benralizumab). Preliminary, encouraging results from the trial (NCT02130882) on the efficacy and safety of the concept of receptor binding-induced antibody-dependent cellular cytotoxicity of eosinophils in iHES have been presented (201). Reslizumab, a different mAB against free IL-5, may be a candidate for the treatment of iHES (191). Therapeutic trials in the pipeline to support the ongoing studies in MLN-TK, CEL, and iHES (**Table 2**), may include TKIs targeting FRGFR, FLT3, or STAT pathways, anti-IgE mABs, perhaps anti-CD52 mABs, and biologicals interfering with IL signaling, all of which have been studied in other diseases (202, 203). All agents may be CS-sparing but are also associated with adverse events that must be included in the benefit–risk assessment to induce longer remissions and disease control in chronic conditions. siglec-8 may be a target for treating iHES and primary eosinophilic conditions in RCTs (92, 204, 205). Still, evidence-based treatment algorithms are missing on eosinophil-directed agents, as are additional guidelines for thrombotic prophylaxis in the target population. Current guidelines in eosinophilia are excellent, describing agents for individualized treatment while taking age, comorbidity, manifestations, and information on cytogenetic and mutational drivers into account (3–7, 17, 18, 20, 58, 61, 136, 137, 145–152) (**Table 1**; **Figure 4**).

QoL is reduced in symptomatic patients with neoplastic or idiopathic eosinophilia and may also be influenced by adverse events to CS or other treatments, which may not outweigh the benefit of symptom reduction (**Table 1**). The development of a disease-specific PRO is important to register and monitor the consequences of eosinophilia and therapeutic interventions. The first important steps have been taken to introduce and validate a questionnaire and to decide on endpoints to be implemented in clinical trials and care (206).

## 10 Conclusion

The observation of eosinophilia in the differential count or blood smear is common and should always merit a reflection as to why it is present. This may, in almost all cases, be readily explained by the diagnostic workup. However, the remaining cases with persistently increased counts and variable symptoms despite adequate interventions, or without a proper diagnosis, may be discussed in a patient-centered MDT. This activity is justified by the pathophysiologic basis and clinical manifestations involving medical and diagnostic specialties. It remains to be demonstrated that the activity in this forum translates to a clinical benefit in patient QoL, overall management, and prognosis. An MDT at a tertiary center can be instrumental to generate data, providing education, and contributing to research. The function supports not only the hematologic subpopulation of patients but also patients with eosinophilia in general.

## Author contributions

GT, CA, HV, and OB designed the project and drafted a major part of the manuscript, **Tables 1**, **2**; **Figures 2**–**5**. HL, JD, KA, CM, AK, TH, SB-O, MM, SJ, and OB created **Supplementary Table 1**. CH and RM-I created **Supplementary Table 2**. MBM created **Figure 1**, and MC created **Table 2**. DEF wrote the section on histiocytosis and IgG4-related disease. All authors contributed to the article and approved the submitted version.
