# Supplementary material for: The multidisciplinary approach to eosinophilia
Source: Front Oncol. 2023 May 18;13:1193730. doi: 10.3389/fonc.2023.1193730 (PMC10232806; doi:10.3389/fonc.2023.1193730)
Supplement: Supplementary file 1 [file Table_1.docx]

SUPPLEMENARY TABLE 1 Manifestations caused by, examinations, and differential diagnoses in patients with eosinophilia.

| **Manifestation** | **Variable symptoms** | **Objective clinical examination^a^** | **Initial laboratory examinations^b^** | **Confirmatory laboratory examinations^c^** | **Differential diagnoses^d^** | **Ref.** |
| --- | --- | --- | --- | --- | --- | --- |
| **Hematological** | B-symptoms (weight loss, low grade fever, night sweats), lymph-adenopathy, hepato-/spleno-megaly, anemia with functional ischemic manifes- tations, bleeding, thrombosis, infec- tions, or acciden- tally discovered | Palpation of lymphadenophaty and organomegaly, hemorrhagic diathesis.  Evaluation of skin changes, e.g. cutaneous lymphoma or mastocytosis | Blood: INR, LDH, urate, creatinine, tryptase, IgA, IgG, IgE, IgM and M-protein | Clonal analysis on blood, and/or bone marrow aspirate +biopsy from organ involvement, imaging (X-ray, US, CT, MRI, PET).  iHES is a diagnosis *per exclusionem* in all manifestations | Acute or chronic leucemia, MPN, MDS, myeloid / lymphoid eosinophilia, malignant lymphoma, GvHD, mastocytosis, histiocytosis | (65-67) |

Legend Table 1: **^a^** always including weight, blood pressure, pulse, respiratory frequency, oxygen saturation; **^b^** proposals for analysis added to initial routine blood samples. And always full cell count (including differential counts), Analysis in blood, otherwise indicated /urine, cerebrospinal fluid); **^c^** specific diagnostic tests to be recommended by specialist, or according to guideline; **^d^** list of differential diagnosis is not prioritized by incidence; Ref. References.

**Abbreviations:**

**Hematological**: CT computerized tomography; GvHD Graft-versus-Host Disease; Ig Immunoglobulin; iHES idiopathic hypereosinophilic syndrome; INR international normalized ratio (coagulation factor II,VII,X); LDH lactate dehydrogenase; MDS myelodysplastic neoplasm; MPN myeloproliferative neoplasm (Philadelphia negative); M-protein monoclonal protein; MRI magnetic resonance imaging; PET positron emission tomography; US ultrasonic imaging; X-ray ordinary imaging (e.g. chest, bone).

| **Manifestation** | **Variable symptoms** | **Objective clinical examination^a^** | **Initial laboratory examinations^b^** | **Confirmatory laboratory examinations^c^** | **Differential diagnoses^d^** | **Ref.** |
| --- | --- | --- | --- | --- | --- | --- |
| **Dermatological** | Prurigo, urticaria, angioedema, erythroderma, macules, papules, plaques, nodules, mucocutaneous ulcers. Cutaneous vascular symp-toms including Raynaud, livedo reticularis, purpura, ulceration and necrosis. Nail splinters.  Vesicles and bullae  B-symptoms.  Symptoms from other organ systems | Dermatological evaluation including differential diagnosis  Evaluation of organomegaly and lymph glands | Skin biopsy (+/- IF )  CRP, ESR, ALAT, LDH, creatinine, Vitamin B12,  IgA,IgG,IgM,IgE, tryptase | Depending on clinical suspicion: KIT D816V mutation peripheral blood, vasculitis screening, blood smear  If organ involvement see other sections | Eosinophilic dermatoses (Eosinophilic cellulitis (Wells syndrome), papulo-erythroderma of Ofuji, granuloma facialis)  Atopic diseases including atopic dermatitis  Cutaneous drug reactions including DRESS  Vasculitis (Churg-Strauss) Immune dysregulation (e.g. Hyper IgE syndromes)  Bullous pemphigoid, scabies, parasitic infections, Sezary Syndrome, mastocytosis,  Gleich syndrome, GvHD | (68) |

Legend Table 1: **^a^** always including weight, blood pressure, pulse, respiratory frequency, oxygen saturation; **^b^** proposals for analysis added to initial routine blood samples. And always full cell count (including differential counts), Analysis in blood, otherwise indicated /urine, cerebrospinal fluid); **^c^** specific diagnostic tests to be recommended by specialist, or according to guideline; **^d^** list of differential diagnosis is not prioritized by incidence; Ref. References.

**Abbreviations:**

**Dermatological**: ALAT alanine amino transferase; CRP C-reactive protein; DRESS drug reaction eosinophilia systemic symptoms; ESR erythrocyte sedimentation rate; GvHD Graft-versus-Host Disease; IF immunofluorescence; Ig Immunoglobulin; LDH lactate dehydrogenase.

| **Manifestation** | **Variable symptoms** | **Objective clinical examination^a^** | **Initial laboratory examinations^b^** | **Confirmatory laboratory examinations^c^** | **Differential diagnoses^d^** | **Ref.** |
| --- | --- | --- | --- | --- | --- | --- |
| **Cardiac** | Shortness of breath with activity or when lying down.  Fatigue and weakness. swelling of the leg and/or abdomen, chest pain, cough, palpitations and embolic events | Cardiac murmurs, Dyspnea, oedema, rapid or irregular heart rhythm.  Echocardiography (Löfflers endocarditis):  LV/RV systolic dysfunction, endomyocardial thickening, with left and right apical mural thrombus formation and posterior mitral leaflet involvement.  Progressive features with restrictive cardiomyopathy and atrioventricular valve regurgitation secondary to subvalvular damage (fibrosis) | ECG nonspecific changes. (ST elevation or non-ST elevation, T wave inversion, atrioventricular block, bundle branch block, ventricular arrhythmia, atrial fibrillation and supraventricular tachycardia).  Biomarkers: Troponine, CK-MB, pro-BNP | Cardiac MRI (if possible with IV gadolinium contrast to improve diagnostic accuracy).  Consider: coronary CT / coronary angiogram (to exclude coronary artery disease).  Consider: endomyo-cardial biopsy (gold standard) | Heart failure. ischemic heart disease, valvular heart disease,  other forms of myocarditis | (69, 70) |

Legend Table 1: **^a^** always including weight, blood pressure, pulse, respiratory frequency, oxygen saturation; **^b^** proposals for analysis added to initial routine blood samples. And always full cell count (including differential counts), Analysis in blood, otherwise indicated /urine, cerebrospinal fluid); **^c^** specific diagnostic tests to be recommended by specialist, or according to guideline; **^d^** list of differential diagnosis is not prioritized by incidence; Ref. References.

**Abbreviations:**

**Cardiac**: CK-MB creatine-kinase myoglobin binding; CT computerized tomography; ECG electrocardiogram; IV intravenous; LV left ventricle; MRI magnetic resonance imaging; pro-BNP pro-Brain Natriuretic Peptide; RV right ventricle.

| **Manifestation** | **Variable symptoms** | **Objective clinical examination^a^** | **Initial laboratory examinations^b^** | **Confirmatory laboratory examinations^c^** | **Differential diagnoses^d^** | **Ref.** |
| --- | --- | --- | --- | --- | --- | --- |
| **Pulmonary** | Dyspnea, cough, fever, pleuritic pain, myalgia, hemoptysis, rhino-sinuitis, B-symptoms, (uncontrolled) asthma, nasal polyposis | Lung auscultation, lung physiology: spirometry with diffusioncapacity or bodypletymography, if asthma suspicion spirometry with reversibility testing and/or provocation tests, 6MWT, saturation, skin affection | Inflammation markers as descri-bed for infectious eosinophilic diseases*.* Aspergillus serology (separate table for parasitic causes).  ANA, ANCA, Quantiferon/ T-spot/ Mantoux test  Radiology: HRCT (detection of patho-gnomonic findings compatible with eosinophilic lung diseases) | BAL: Flowcytometry (detection of eosino-philia). Culture and microscopy for fungi, PCR for Pneumocystis Jirovecii, TB, NTM  Tissue: TBB or TBCB (detection of organ-related eosinophilia and pathognomonic histological findings) | Primary parenchymal eosinophilic lung diseases: iAEP, iCEP, HES, iHypereosinophilic BO, pulmonary EGPA (see “Rheumatological”)  Primary airway-related eosinophilic lung diseases: Eosinophilic asthma, eosinophilic bronchitis  Secondary airway-related eosinophilic lung diseases: Asthma, ABPA, ABPM  Secondary parenchymal eosinophilic lung diseases: CPA, TB, NTM, PCP, HIV, drugs, IPF, lung cancer, PLCH | (71-73) |

Legend Table 1: **^a^** always including weight, blood pressure, pulse, respiratory frequency, oxygen saturation; **^b^** proposals for analysis added to initial routine blood samples. And always full cell count (including differential counts), Analysis in blood, otherwise indicated /urine, cerebrospinal fluid); **^c^** specific diagnostic tests to be recommended by specialist, or according to guideline; **^d^** list of differential diagnosis is not prioritized by incidence; Ref. References.

**Abbreviations:**

**Pulmonary**: ABPA Allergic bronchopulmonary aspergillosis; ABPM Allergic bronchopulmonary mycosis; ANA anti-nuclear antibodies; ANCA Anti-neutrophil cytoplasmic autoantibodies; BAL Bronchoalveolar lavage; BO Bronchiolitis obliterans; (C)OP (Cryptogenic) organising pneumonia; CPA Chronic pulmonary aspergillosis; EGPA Eosinophilic granulomatosis with polyangiitis; HES hypereosinophilic syndrome; HIV human immunodeficiency virus; HRCT high resolution computed tomography; iAEP Idiopathic acute eosinophilic pneumonia; iCEP Idiopathic chronic eosinophilic pneumonia; IPF Idiopathic pulmonary fibrosis; NTM Non-tubercolous mycobacteria; PCP Pneumocystis Carini (now Jirovecii) Pneumonia; PCR polymerase chain reaction; PLCH Pulmonary Langerhans cell histiocytosis; TB Tuberculosis; TBB Transbronchial biopsy; TBCB Transbronchial cryobiopsy; 6MWT six minute walking test.

| **Manifestation** | **Variable symptoms** | **Objective clinical examination^a^** | **Initial laboratory examinations^b^** | **Confirmatory laboratory examinations^c^** | **Differential diagnoses^d^** | **Ref.** |
| --- | --- | --- | --- | --- | --- | --- |
| **Rheumatological** | B-symptoms. Myo-arthralgia. Uncontrolled asthma. Chronic rhinosinuitis and nasal polypose.  Glomerulo nephritis.  Pericarditis and cardiac rhythm abnormalities. Mononeuritis multiplex (sensory, motoric dysfunction) | Lymphadenopathy  Palpable purpura or urticaria | Blood: albumin, CRP, IgG, IgA, IgM and IgE. ANCA  Urine analysis with microscopic examination of urinary sediment  Radiology:  X-ray of the chest and HRCT  Sinus-CT  (Cardiac MRI)  ECG, Echocardiography | Tissue biopsy with eosinophilic infiltration, granulomatosis and necrotizing vasculitis from e.g. Lung, Skin  Nasal or Kidney  Nerve conduction and EMG | EPGA – multiorgan system disease  Differential diagnosis are the other ANCA-associated vasculitis GPA and MPA | (74, 75) |

Legend Table 1: **^a^** always including weight, blood pressure, pulse, respiratory frequency, oxygen saturation; **^b^** proposals for analysis added to initial routine blood samples. And always full cell count (including differential counts), Analysis in blood, otherwise indicated /urine, cerebrospinal fluid); **^c^** specific diagnostic tests to be recommended by specialist, or according to guideline; **^d^** list of differential diagnosis is not prioritized by incidence; Ref. References.

**Abbreviations:**

**Rheumatological**: ANCA anti-neutrophil cytoplasmic antibodies; CRP C-reactive protein; CT computerized tomography; ECG electrocardiogram; EMG electromyography; EPGA Eosinophilic granulomatosis with polyangiitis; GPA granulomatosis with polyangiitis; HRCT high resolution computed tomography; Ig immunoglobulin; MPA microscopic polyangiitis; MRI magnetic resonance imaging; ; X-ray ordinary imaging (e.g. chest, bone).

| **Manifestation** | **Variable symptoms** | **Objective clinical examination^a^** | **Initial laboratory examinations^b^** | **Confirmatory laboratory examinations^c^** | **Differential diagnoses^d^** | **Ref.** |
| --- | --- | --- | --- | --- | --- | --- |
| **Oto rhino laryngology** | Nasal obstruction, nasal secretion, Loss of sense of smell, pain and/or pressure over sinus  Ear pain/ earfullness | Test of nasal breathing  Nasal endoscopy  Test of sense of smell  Otoscopy  Examination of the throat  Symptom score using the PROM SNOT-22 | Biopsy of nasal polyps with count of eosinophilic cells Total IgE | CT-scanning of the nasal sinus | Chronic Rhinosinusitis with or without nasal polyposis  Eosinophilic GPA  CRS wNP is most often a type 2 inflammation in the nasal mucosa. In rare cases the disease is part of primary eosinophilia | (76, 77) |
| **Gastro- enterological and liver** | Dysphagia. Pain. Nausea and vomiting. Diarrhea. Weight loss. Obstruction. Abdominal distention, jaundice | No specific findings, or weight loss and signs of malnutrition.  Signs of liver failure (ascites, icterus, spider naevi, palmar erythema) | Nutrition panel.  Liver function tests. Autoimmune liver serology | Endoscopy with mucosal biopsies according to presenting symptoms.  Imaging with US and CT  Ascites cytology and liver biopsy. MRCP | Infection. Drug reaction. Inflammatory bowel disease.  Toxic hepatitis. Autoimmune hepatitis. Sclerosing cholangitis | (78) |

Legend Table 1: **^a^** always including weight, blood pressure, pulse, respiratory frequency, oxygen saturation; **^b^** proposals for analysis added to initial routine blood samples. And always full cell count (including differential counts), Analysis in blood, otherwise indicated /urine, cerebrospinal fluid); **^c^** specific diagnostic tests to be recommended by specialist, or according to guideline; **^d^** list of differential diagnosis is not prioritized by incidence; Ref. References.

**Abbreviations:**

**Oto rhino laryngology:**  CRS wNP Chronic Rhinosinusitis with Nasal Polyposis; CT computerized tomography; GPA granulomatosis with polyangiitis; Ig immunoglobulin; PROM (Patient reported outcome measure) SNOT-22 ( Sino-Nasal Outcome Test 22 items).

**Gastroenterological and liver:** CT computerized tomography; MRCP magnetic resonance cholangiopancreatography; US ultrasound.

| **Manifestation** | **Variable symptoms** | **Objective clinical examination^a^** | **Initial laboratory examinations^b^** | **Confirmatory laboratory examinations^c^** | **Differential diagnoses^d^** | **Ref.** |
| --- | --- | --- | --- | --- | --- | --- |
| **Neurological** | Cerebral throm-bosis, mostly arterial with paresis. Visual dysfunction. Encephalopathy, particularly cognitive and / or upper neuron paresis. Peripheral neuropathies, sym metric or not, sen sory or motoric or both. Mononeuritis multiplex | Consciousness, cranial nerve testing, neck stiffness, muscle function (paresis), deep tendon reflexes, sensibility, coordination of movements, cognitive testing, speech | Blood glucose, ionized calcium, thyroid test, liver enzymes and INR, vitamin B6 and B12    CT cerebrum, lumbar puncture (incl cell counts, protein, microscopy and culture) | MRI cerebrum and/or medulla spinalis. Electro diagnostic analysis of nerve and muscle function, EEG, Nerve or brain biopsy.  Autoimmune anti bodies ANA and ANCA,  CSF: oligoclonal bands in CSF and other specific tests as indicated | Apoplexia, disseminated sclerosis, Guillain-Barré syndrome, intoxication (alcoholism, medication, metal, metabolic), uncontrolled diabetes, infectious meningitis, increased intracranial pressure, tumor – malignant or benign, EGPA, other ANCA-associated vasculitis GPA and MPA | (79, 80) |

Legend Table 1: **^a^** always including weight, blood pressure, pulse, respiratory frequency, oxygen saturation; **^b^** proposals for analysis added to initial routine blood samples. And always full cell count (including differential counts), Analysis in blood, otherwise indicated /urine, cerebrospinal fluid); **^c^** specific diagnostic tests to be recommended by specialist, or according to guideline; **^d^** list of differential diagnosis is not prioritized by incidence; Ref. References.

**Abbreviations:**

**Neurological**: ANA Anti-nuclear antibody, ANCA anti-neutrophil cytoplasmic antibodies; CSF cerebrospinal fluid; CT computerized tomography; EEG electroencephalogram; EGPA Eosinophilic granulomatosis with polyangiitis; GPA granulomatosis with polyangiitis; INR international normalized ratio (coagulation factor II,VII,X); MPA microscopic polyangiitis; MRI magnetic resonance imaging.

| **Manifestation** | **Variable symptoms** | **Objective clinical examination^a^** | **Initial laboratory examinations^b^** | **Confirmatory laboratory examinations^c^** | **Differential diagnoses^d^** | **Ref.** |
| --- | --- | --- | --- | --- | --- | --- |
| **Nephro-urological** | Pain (flank, lower abdominal), dysuria, hematuria, urine retention, symptoms of renal failure, fever | Abdominal palpation (pain-characteristics, mass) | Blood: creatinine, clearance, blood urea nitrogen, PSA in males; urine analysis (dipstick, quantitative protein and volume, eosinophils in urine), culture and microscopy for microorganisms  Imaging: US, CT, MRI, nuclear scan | Cysto-uretero-scopy, renal biopsy | Glomerulopathy – autoimmune or infectious, acute tubular necrosis. EGPA, GPA, MPA, IgG4-related disease, Erdheim-Chester disease, sarcoidosis, DRESS, parasitic diseases, eosinophilic cystitis, dialysis-associated eosinophilia, renal malignancy | (81, 82) |

Legend Table 1: **^a^** always including weight, blood pressure, pulse, respiratory frequency, oxygen saturation; **^b^** proposals for analysis added to initial routine blood samples. And always full cell count (including differential counts), Analysis in blood, otherwise indicated /urine, cerebrospinal fluid); **^c^** specific diagnostic tests to be recommended by specialist, or according to guideline; **^d^** list of differential diagnosis is not prioritized by incidence; Ref. References.

**Abbreviations:**

**Nephro-urological**: CT computerized tomography; DRESS drug reaction eosinophilia systemic symptoms; EGPA Eosinophilic granulomatosis with polyangiitis; GPA granulomatosis with polyangiitis; ); Ig immunoglobulin; MPA microscopic polyangiitis; MRI magnetic resonance imaging; PSA Prostate Specific Antigen; US ultrasound.

| **Manifestation** | **Variable symptoms** | **Objective clinical examination^a^** | **Initial laboratory examinations^b^** | **Confirmatory laboratory examinations^c^** | **Differential diagnoses^d^** | **Ref.** |
| --- | --- | --- | --- | --- | --- | --- |
| **Congenital**  **FE: IEI: inborn errors**  **Familial eosinophilia of immunity** | IEI: Atopy, recurrent infections (bacterial, viral, fungal), enteropathy, auto-inflammation/-immunity, failure to thrive  FE: no specific symptoms, no increased infectious burden or findings consistent with atopy or immune dysregulation | IEI: Familial predis-position, eczema, skin abscesses, adenopathy, eosinophilia, lympho-penia, elevated serum IgE, skeletal abnormali-ties, syndromic features  FE: End organ damage due to eosinophilia not consistently observed | IEI: Red and white blood count, serum immunoglobulins, vaccination status, lymphocyte surface markers  FE: persistent eosinophilia > 1.5 x 10^9^/L, serum immunoglobulins, skin prick test, allergen specific IgE, and diagnostic work-up: e.g. infec-tious diseases, pul-monary, rheumato-logy, hematological | IEI: Genetic sequencing for monogenetic germline IEI variants associated with eosinophilia. Functional molecular/cellular studies, serum cytokines  FE: Genetic sequencing if available serum cytokines/ eosinophil granule proteins, eosinophil activation markers (CD25, CD69, HLA-DR). Classified as autosomal dominant disease, secondary to dysregulated IL-5 production | IEI: DN STAT3 Job’s syndrome, AR DOCK8, XL WAS, Omenn syndrome (AR RAG1/2, XL IL2RG, AR/AD IL7R), AR IL6R deficiency, IPEX FOXP3, AD GOF JAK1  FE: iHE or iHES, primary or secondary eosinophilia | IEI:  (83, 84)  FE:  (85, 86) |

Legend Table 1: **^a^** always including weight, blood pressure, pulse, respiratory frequency, oxygen saturation; **^b^** proposals for analysis added to initial routine blood samples. And always full cell count (including differential counts), Analysis in blood, otherwise indicated /urine, cerebrospinal fluid); **^c^** specific diagnostic tests to be recommended by specialist, or according to guideline; **^d^** list of differential diagnosis is not prioritized by incidence; Ref. References.

**Abbreviations:**

**Congenital causes**: AD autosomal dominant; AR autosomal recessive; CD cluster of differentiation; DN dominant negative; DOCK8 dedicator of cytokinesis 8; FE familial eosinophilia; FOXP3 forkhead box P3; GOF gain-of-function; HLA-DR human leukocyte antigen – DR isotype; IEI inborn errors of immunity; Ig immunoglobulin; iHES idiopathic hypereosinophilic syndrome; IL2RG interleukin 2 receptor subunit gamma; IL5 interleukin 5; IL6R interleukin 6 receptor; IL7R interleukin 7 receptor; IPEX immune dysregulation, polyendocrinopathy, enteropathy, X-linked syndrome; JAK1 Janus kinase 1; RAG1/2 recombination activating 1/2; STAT3 signal transducer and activator of transcription 3; WAS Wiskott–Aldrich syndrome; XL X-linked.
