# Supplementary material for: The multidisciplinary approach to eosinophilia
Source: Front Oncol. 2023 May 18;13:1193730. doi: 10.3389/fonc.2023.1193730 (PMC10232806; doi:10.3389/fonc.2023.1193730)
Supplement: Supplementary file 2 [file Table_2.docx]

SUPPLEMENTARY TABLE 2 Common infectious causes of eosinophilia due to parasites.

| **Infectious organism** | **Classification** | **Other clinical symptoms** | **Main geographic areas of distribution** | **Diagnostic tests** |
| --- | --- | --- | --- | --- |
| **Gastrointestinal symptoms and eosinophilia** | | | | |
| Anisakis spp. | Nematode | Pruritus of the posterior oropharynx, acute epigastric pain, severe abdominal pain, urticaria | Japan, Europe | Endoscopy (visualize worm) |
| Ancylostoma duodenale and Necator americanus (hookworm)^1^ | Nematode | Asymptomatic, parasitic Loeffler’s syndrome^1^, abdominal pain, diarrhea, iron-deficiency anemia | Sub-Saharan Africa, Latin America, Asia, Western Pacific | Stool microscopy (eggs) |
| Ascaris lumbricoides | Nematode | Asymptomatic, parasitic Loeffler’s syndrome^1^, urticaria, abdominal pain, diarrhea, biliary obstruction | Sub-Saharan Africa, Latin America, Asia, Western Pacific | Stool microscopy (eggs) |
| Gnathostoma spp. | Nematode | Asymptomatic, fever, abdominal pain, urticaria.  Migrating larvae cause localized subcutaneous swellings | Southeast Asia, Latin America | Microscopy of cutaneous biopsy (larvae) |
| Strongyloides stercoralis ^1,2^ | Nematode | Parasitic Loeffler’s syndrome^1^, larva currens, abdominal pain, diarrhea, hyperinfestation syndrome | Tropical and subtropical areas | Stool microscopy (larvae)  Serology |
| Toxocara canis and Toxocara cati | Nematode | Asymptomatic. Visceral larva migrans: abdominal pain, hepatosplenomegaly, cough, bronchospasm, asthma, fever. Ocular toxocariasis: posterior uveitis | Worldwide, mostly children in tropical and subtropical areas | Serology |
| Trichuris trichiura (whipworm) | Nematode | Asymptomatic, diarrhea, dysentery, anemia, rectal prolapse | Tropical and subtropical areas | Stool microscopy (eggs) |
| Clonorchis spp. | Trematode (liver fluke) | Asymptomatic, fever, right upper quadrant pain, hepatomegaly, urticaria | Asia | Stool microscopy (eggs) |
| Fasciola hepatica | Trematode (liver fluke) | Fever, right upper quadrant pain, hepatomegaly, jaundice, biliary obstruction, urticaria | Worldwide (highest incidence in South America) | Stool microscopy (eggs)  Serology |
| Opisthorchis spp. | Trematode (liver fluke) | Asymptomatic, fever, right upper quadrant pain, hepatomegaly, urticaria | Asia and states of the former Soviet Union | Stool microscopy (eggs) |
| Acute schistosomiasis  (Katayama fever) | Trematode (blood fluke) | Fever, urticarial rash, malaise, myalgia, diarrhea, cough, abdominal pain, and headache, swimmer’s itch | Africa, Latin America, the Middle East, southern China, Asia, Western Pacific, Corsica^3^ | Clinical diagnosis  Serology  PCR (serum) |
| Chronic schistosomiasis  (S. mansoni, intercalatum, japonicum, mekongi) | Trematode (blood fluke) | Asymptomatic, abdominal pain, ﻿periportal fibrosis, pre-sinusoidal portal hypertension, hepatosplenomegaly, varices | Africa, Latin America, the Middle East, southern China, Southeast Asia, Western Pacific | Stool microscopy (eggs)  Schistosoma serology  Microscopy of rectal biopsy (eggs)  PCR (feces) |
| Diphyllobothrium latum | Cestode (fish tapeworm) | Asymptomatic, diarrhea, B12 deficiency | Europe, states of the Former Soviet Union, North America, Asia | Stool microscopy (eggs) |
| Echinococcus (E. granulosus (hydatid cyst), E multilocularis) | Cestode | Asymptomatic, right upper quadrant pain, hepatomegaly | Africa, Europe, Asia, the Middle East, Latin America | Abdominal US  Thorax/abdomen CT scan  Serology |
| Hymenolepis nana | Cestode (dwarf tapeworm) | Asymptomatic, abdominal pain | Tropical and subtropical areas | Stool microscopy (eggs) |
| Taenia solium and T. saginata (Intestinal taeniasis) | Cestode | Asymptomatic, abdominal pain | Worldwide | Stool microscopy (eggs) or taenia segments in a stool sample |
| Cystoisospora belli (formerly Isospora belli) | Protozoa | Abdominal pain, diarrhea | Tropical and subtropical areas | Stool microscopy (oocysts) |
| **Pulmonary symptoms and eosinophilia** | | | | |
| Ancylostoma duodenale and Necator americanus | Nematode | Asymptomatic, parasitic Loeffler’s syndrome^1^, abdominal pain, diarrhea, iron-deficiency anemia | Sub-Saharan Africa, Latin America, Asia, Western Pacific | Stool microscopy (eggs) |
| Ascaris lumbricoides | Nematode | Asymptomatic, parasitic Loeffler’s syndrome^1^, urticaria, abdominal pain, diarrhea, biliary obstruction | Sub-Saharan Africa, Latin America, Asia, Western Pacific | Stool microscopy (eggs) |
| Dirofilaria immitis (dog heartworm) | Nematode | Asymptomatic, fever, chest pain, cough, hemoptysis | Mediterranean region, US, Eastern Europe, Central Asia | Worm detection in biopsy  Serology  PCR |
| Strongyloides stercoralis ^1,2^ | Nematode | Parasitic Loeffler’s syndrome^1^, larva currens, abdominal pain, diarrhea, hyperinfestation syndrome | Tropical and subtropical areas | Stool microscopy (larvae)  Serology |
| Wuchereria bancrofti or Brugia malayi | Nematode | Tropical pulmonary eosinophilia, lymphedema of the extremities, hydrocele | Sub-Saharan Africa, Southeast Asia, Western Pacific | Microfilaria in night time blood smear  Filaria serology  PCR |
| Paragominus westermani | Trematode (lung fluke) | Fever, abdominal pain, pleuritic pain, hemoptysis, urticaria | Southeast Asia, Western Pacific, Japan | Sputum (or stool) microscopy (eggs)  Serology |
| **Allergy or skin/soft tissue symptoms and eosinophilia** | | | | |
| Anisakis spp. | Nematode | Pruritus in the oropharynx, abdominal pain, vomiting, urticaria | Japan, Europe | Endoscopy |
| Ascaris lumbricoide | Nematode | Asymptomatic, parasitic Loeffler’s syndrome^1^, urticaria, abdominal pain, diarrhea, biliary obstruction | Sub-Saharan Africa, Latin America, Asia, Western Pacific | Stool microscopy (eggs) |
| Dirofilaria repens | Nematode | Subcutaneous swellings (migration of the worm) | Mediterranean region, US, Eastern Europe, Central Asia | Worm detection in biopsy  Serology  PCR |
| Gnathostoma spp. | Nematode | Asymptomatic, fever, abdominal pain, urticaria.  Migrating larvae cause localized subcutaneous swellings | Southeast Asia, Latin America | Microscopy of cutaneous biopsy (larvae) |
| Loa loa | Nematode | Calabar swellings, eye worm | West and Central Africa | Microfilaria in mid-day blood smear  Filaria serology  Identification of the eyeworm  PCR |
| Mansonella spp. | Nematode | Asymptomatic, pruritus, urticaria, subcutaneous swellings, abdominal pain, pleuritis | Africa, Latin America | Microfilaria in blood smear  Skin snip  Serology  PCR |
| Onchocerca volvulus  (River blindness) | Nematode | Severe pruritus, papular onchodermatitis, ocular changes, blindness, subcutaneous nodules | Sub-Saharan Africa, Latin America, and Middle East | Skin snip  Adult worm in skin nodules  Slit lamp^4^  Serology  PCR |
| Strongyloides stercoralis ^1,2^ | Nematode | Parasitic Loeffler’s syndrome^1^, larva currens, abdominal pain, diarrhea, hyperinfestation syndrome | Tropical and subtropical areas | Stool microscopy (larvae)  Serology |
| Wuchereria bancrofti or Brugia malayi (Lymphatic filariasis) | Nematode | Tropical pulmonary eosinophilia, lymphedema of the extremities, hydrocele | Sub-Saharan Africa, Southeast Asia, Western Pacific | Microfilaria in night-time blood smear  Filaria serology  PCR |
| Clonorchis spp. | Trematode (liver fluke) | Asymptomatic, fever, right upper quadrant pain, hepatomegaly, urticaria | Asia | Stool microscopy (eggs) |
| Opisthorchis spp. | Trematode (liver fluke) | Asymptomatic, fever, right upper quadrant pain, hepatomegaly, urticaria | Asia and states of the former Soviet Union | Stool microscopy (eggs) |
| Paragominus westermani | Trematode (lung fluke) | Fever, abdominal pain, pleuritic pain, urticaria | Southeast Asia, Western Pacific, Japan | Sputum (or stool) microscopy (eggs) Serology |
| **Myositis and eosinophilia** | | | | |
| Trichinella spiralis | Nematode | Fever, periorbital or facial edema | Worldwide | Serology, muscle biopsy |
| Sarcocystis spp. | Protozoa | Fever, headache, diarrhea, myositis of the muscles of mastication | Malaysia | MRI, muscle biopsy |
| **Genitourinary symptoms and eosinophilia** | | | | |
| Chronic Schistosomiasis (S. haematobium) | Trematode (blood fluke) | Hematuria, bladder fibrosis/calcifications, obstructive uropathy, bladder cancer | Africa, the Middle East, Corsica^3^ | Urine microscopy (eggs)  Schistosoma serology  PCR in urine |
| **Eosinophilic meningitis** | | | | |
| Angiostrongylus cantonensis | Nematode | Eosinophilic meningitis | Southeast Asia, Western Pacific, Caribbean, Africa | Eosinophilic pleocytosis  PCR (CSF)  MRI |
| Gnathostoma spinigerium | Nematode | Eosinophilic meningoencephalitis, myelitis | Southeast Asia, China, and Japan | Eosinophilic pleocytosis  MRI |
| **Other non-parasitic infections** | | | | |
| Allergic bronchopulmonary aspergillosis | Fungal | Asthma, recurrent exacerbations | Worldwide | Serology  Culture of sputum  HRCT scan of the thorax |
| Coccidioidomycosis | Fungal | Pneumonia, fever, desert rheumatism, erythema nodosum, erythema multiforme | US, Latin America | Serology  Culture of sputum |

Legend Table 2: abbreviations: CSF, cerebrospinal fluid; CT scan, Computed Tomography scan; HRCT, High-Resolution Computed Tomography scan; MRI; Magnetic Resonance Imaging; PCR, polymerase chain reaction; spp, species; US, ultrasonography.

^1^ They can cause parasitic Loeffler’s syndrome or transient, self-limiting eosinophilic pneumonia due to larval migration through the lungs.

^2^ Most common infectious cause of eosinophilia. It is a soil-transmitted helminth with an auto-infective cycle.

^3^ Ongoing transmission has been identified in Cavu River in Corsica.

^4^ Identification of the larvae in the anterior part of the eye (slit lamp)

It is important to follow local microbiological guidelines regarding the proper collection and sending of biological material for diagnostics

References (87, 88).
